# Regulation of VEGFR3 signaling in lymphatic endothelial cells

**DOI:** 10.3389/fcell.2025.1527971

**Published:** 2025-02-13

**Authors:** Kevin G. Kuonqui, Adana-Christine Campbell, Bracha L. Pollack, Jinyeon Shin, Ananta Sarker, Stav Brown, Hyeung Ju Park, Babak J. Mehrara, Raghu P. Kataru

**Affiliations:** Plastic and Reconstructive Surgery Service, Department of Surgery, Memorial Sloan Kettering Cancer Center, New York, NY, United States

**Keywords:** VEGFC, lymphangiogenesis, lymphatics, lymphedema, VEGFR3, LECs

## Abstract

The receptor tyrosine kinase vascular endothelial growth factor (VEGF) receptor 3 (VEGFR3) is the principal transmembrane receptor responsible for sensing and coordinating cellular responses to environmental lymphangiogenic stimuli in lymphatic endothelial cells (LECs). VEGFC and D (VEGFC/D) function as the cognate ligands to VEGFR3 by stimulating autophosphorylation of intracellular VEGFR3 tyrosine kinase domains that activate signal cascades involved in lymphatic growth and survival. VEGFR3 primarily promotes downstream signaling through the phosphoinositide 3-kinase (PI3K) and Ras signaling cascades that promote functions including cell proliferation and migration. The importance of VEGFR3 cascades in lymphatic physiology is underscored by identification of dysfunctional VEGFR3 signaling across several lymphatic-related diseases. Recently, our group has shown that intracellular modification of VEGFR3 signaling is a potent means of inducing lymphangiogenesis independent of VEGFC. This is important because long-term treatment with recombinant VEGFC may have deleterious consequences due to off-target effects. A more complete understanding of VEGFR3 signaling pathways may lead to novel drug development strategies. The purpose of this review is to 1) characterize molecular mediators of VEGFC/VEGFR3 downstream signaling activation and their functional roles in LEC physiology and 2) explore molecular regulation of overall VEGFR3 expression and activity within LECs.

## Introduction

Activation of vascular endothelial growth factor (VEGF) receptor 3 (VEGFR3) intracellular signaling cascades by VEGF-C (VEGFC) is essential for initial lymphatic formation during embryogenesis and postnatal lymphangiogenesis by regulating lymphatic endothelial cell (LEC) differentiation, proliferation, migration, and function (Zhang et al., 2010; Mäkinen et al., 2001a; Mäkinen et al., 2001b). During embryogenesis, LECs are thought to differentiate from the mesenchyme and originate from the cardinal vein (Yang et al., 2012). Postnatally, new lymphatic growth is primarily driven by VEGFR3-mediated proliferation of LECs originating from pre-existing lymphatic networks. However, bone marrow-derived endothelial progenitors may also contribute to postnatal lymphangiogenesis (Stanczuk et al., 2015; PARK et al., 2011). Heterozygous VEGFC gene deletion during embryogenesis results in hypoplastic dermal lymphatics and lymphedema in mouse pups that persists into adulthood, highlighting the importance of this ligand in regulating proper lymphatic function (Karkkainen et al., 2004). Following ligand-mediated engagement of membrane-bound VEGFR3, receptor homodimerization (VEGFR3-VEGFR3) or heterodimerization (VEGFR2-VEGFR3) catalytically induce intracellular tyrosine kinase autophosphorylation that recruits cytoplasmic secondary messengers (Koch et al., 2011; Alam et al., 2004). VEGFR2-VEGFR3 heterodimer formation preferentially promotes phosphoinositide 3-kinase (PI3K)–dependent activation of protein kinase B (AKT), while VEGFR3 homodimers preferentially stimulate p42/p44 mitogen–activated protein kinase (MAPK, also known as ERK1/2) activation (DENG et al., 2015; Mäkinen et al., 2001b). AKT and ERK serve as the principal downstream effectors of the VEGFR3 axis; however, activation of other effectors, such as the c-Jun N-terminal kinase (JNK) axis, is also important (Koch et al., 2011; Mäkinen et al., 2001b). This review will focus on outlining knockout or gain of function experiments revealing what is known about VEGFR3 activation, intracellular signal transduction, and expression under normal conditions. Further information correlating known alterations in VEGFR3 regulation in clinically relevant disease states is outside the scope of this review and are explored in more detail by another review recently published by our group (Kuonqui et al., 2023).

Three distinct VEGFR orthologs have been described: VEGFR1, VEGFR2, VEGFR3 (Galland et al., 1993; Aprelikova et al., 1992). VEGFR1 is expressed on blood endothelial cells (BECs) and myeloid cells, while VEGFR2 and VEGFR3 are most abundantly expressed on BECs and LECs, respectively (Koch et al., 2011; Olsson et al., 2006; Kerber et al., 2008; Murakami et al., 2008; Muramatsu et al., 2010). Structurally, VEGFRs are comprised of five main regions: 1) extracellular Ig-like ligand–binding domains, 2) a transmembrane domain, 3) a juxtamembrane domain, 4) a split tyrosine kinase domain, and 5) a C-terminal tail (Koch et al., 2011; Pajusola et al., 1992). Of note, VEGFR3 uniquely undergoes proteolytic cleavage of its fifth extracellular Ig-like-ligand-binding domain, resulting in the formation of two disulfide bridge-linked polypeptide chains (Pajusola et al., 1994). The human *Fms-like tyrosine 4* (*FLT4*) gene can encode the production of 5.8 kb and 4.5 kb VEGFR3 mRNA transcripts via alternative splicing, with the longer isoform being most commonly found in the majority of tissues (Pajusola et al., 1992; Pajusola et al., 1993). The longer isoform (FLT41) undergoes translation to form a 195 kDa VEGFR3 polypeptide precursor that is 65 residues longer at its C-terminus compared to its shorter counterpart (FLT4s), which subsequently undergoes proteolytic cleavage to form a functionally mature VEGFR3 transmembrane protein (Koch et al., 2011; Pajusola et al., 1992; Pajusola et al., 1993). Within the long VEGFR3 isoform, the additional residues are thought to confer additional autophosphorylation sites necessary for intracellular adapter protein recruitment, leading to differing functional capacities between the two isoforms (Borg et al., 1995; Hughes, 2001; Fournier et al., 1996). In contrast, mice have been found to produce a single VEGFR3 mRNA isoform (Hughes, 2001).

The human *VEGFC* gene encodes an unprocessed 58 kDa vascular endothelial growth factor C (VEGFC) precursor polypeptide, which contains N-terminal and C-terminal domains which must be proteolytically cleaved to allow proper functional interactions with VEGFR3 and VEGFR2 to promote lymphangiogenesis (Joukov et al., 1996; Joukov et al., 1997). Intracellularly, proprotein convertases, such as furin, cleave C-terminal domains from unprocessed polypeptide precursors to form pro-VEGFC molecules, which can only bind VEGFR3 (Siegfried et al., 2003). Extracellularly, Collagen- and calcium-binding epidermal growth factor domains 1 (CCBE1) indirectly promotes further proteolytic VEGFC cleavage via activation of the A disintegrin and metalloprotease with thrombospondin motifs-3 (ADAMTS3) metalloprotease, ultimately resulting in removal of the N-terminal domain to produce a mature 21/23 kDa VEGFC ligand, which can bind both VEGFR2 and VEGFR3 (Jeltsch et al., 2014; Bui et al., 2016).

VEGFR-binding domains on VEGFC ligand mediate binding to both VEGFR3 and VEGFR2 tyrosine kinase receptors (Joukov et al., 1996). VEGFC interacts with Ig-like loops 1 and 2 on the VEGFR3 extracellular ligand-binding region, while VEGFC interacts with Ig-like loop 2 on the VEGFR2 extracellular ligand-binding region (Jeltsch et al., 2006). Following ligand binding, VEGFR tyrosine kinase receptors dimerize to initiate an intracellular signal transduction cascade. Subsequent engagement of extracellular Ig-like loops 5 and 7 have been implicated in facilitating functional VEGFR3 homodimerization and activation (Leppänen et al., 2013). In LECs, VEGF-C can induce receptor homodimerization (VEGFR3-VEGFR3) or heterodimerization (VEGFR2-VEGFR3). VEGFC activation of the intracellular tyrosine kinase region stimulates autophosphorylation of tyrosine residues, leading to the recruitment of cytoplasmic adapter proteins that activate downstream signaling cascades (Koch et al., 2011; Fournier et al., 1999; Fournier et al., 1996). Notably, VEGFR3 homodimerization leads to autophosphorylation of 5 tyrosine residues (Tyr^1230,^ Tyr^1231^, Tyr^1265^, Tyr^1337^, Tyr^1363^) on each receptor, while VEGFR3-VEGFR2 heterodimerization fails to phosphorylate 2 tyrosine residue sites (Tyr^1337^, Tyr^1363^) on VEGFR3, thereby suggesting differential downstream signaling activities, which are further explored in subsequent sections related to intracellular VEGFR3 signaling pathways (Dixelius et al., 2003).

### Initiation and Co-modulation of transmembrane VEGFR3 signaling

#### Regulation of VEGFR3 signaling by co-receptors and membrane-associated proteins

**FIGURE 1:**
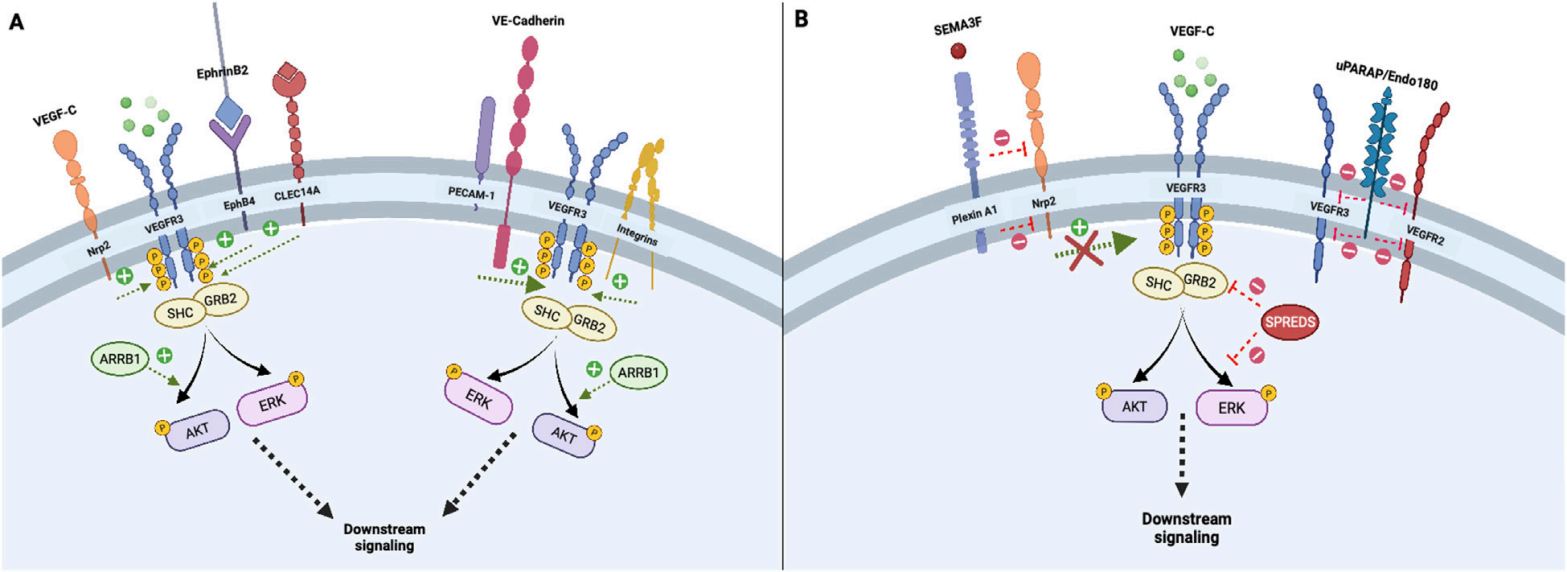
Initiation and co-modulation of transmembrane VEGFR3 signaling; Transmembrane receptors and intracellular adapter proteins regulate VEGFR3 tyrosine kinase domain activation and signal transduction. Neuropilin-2 and EphB4 receptors promote internalization of VEGF-C/VEGFR3 complexes, which is necessary for downstream signal propagation **(A)**. In contrast, Plexin A1 signaling attenuates VEGFR3 *via* Neuropilin-2 inhibition, while uPARAP inhibits VEGFR2-VEGFR3 heterodimer formation, thereby fine-tuning lymphangiogenic activity **(B)**. Other transmembrane proteins including the PECAM-1/VE-Cadherin mechanosensory complex and membrane integrins, enhance downstream VEGFR3 signaling **(A)**. Intracellular adapters including Shc and Grb2 activate principal downstream VEGFR3 effectors AKT and ERK, which can be enhanced or attenuated by cytoplasmic proteins ARRB1 and SPREDS **(A, B)**.

Several co-receptors and membrane–associated proteins increase VEGFR3 signaling or activation, which are summarized in Figure 1A. For example, interactions between VEGFC and co-receptor neuropilin-2 (Nrp2) directly engage VEGFR3 to increase downstream signal transduction, supporting functions such as tip cell activation during lymphatic network sprouting (Xu et al., 2010; Parker et al., 2015). Likewise, EphrinB2-EphB4 receptor activity in LECs augments VEGFR3-dependent AKT and ERK activation by facilitating growth factor/receptor internalization (Wang et al., 2010; Wu et al., 2019). Urokinase plasminogen activator receptor-associated protein (uPARAP) inhibits VEGFR2-VEGFR3 heterodimer formation and VEGFR2 signaling, thereby favoring VEGFR3 activation and cellular migration in response to VEGFC (Durré et al., 2018). Angiopoietin 2/Tie receptor axis signaling also induces downstream activation of the PI3K/AKT pathway, which promotes LEC responsiveness to VEGFC stimulation by maintaining VEGFR3 cell surface availability (Korhonen et al., 2022). Specifically, the Ang2/Tie/PI3K signaling axis increases cell surface VEGFR3 expression by augmenting early endosomal VEGFR3 recycling to the plasma membrane and circumventing late endosomal degradation pathways. (Korhonen et al., 2022) (Figure 1A).

Other co-receptors or membrane-associated proteins decrease VEGFR3 activation. In cultured human dermal LECs (but not *in vivo* LECs), vascular endothelial phosphotyrosine phosphatase (VE-PTP) attenuates VEGFR3 signaling by mediating dephosphorylation of its tyrosine kinase domains (DENG et al., 2015; Souma et al., 2018). Deng *et al* also found that VE-PTP knockdown in cultured LECs promotes increased VEGFC-induced VEGFR3 internalization*,* illustrating a receptor trafficking-based mechanism by which VE-PTP inhibits VEGFR3 signal transduction (DENG et al., 2015). In contrast to increased VEGFR3 signaling resulting from binding of Nrp2 by VEGFC, binding of Nrp2 by semaphorin 3F (SEMA3F) or PlexinA1 ligands dampens VEGFR3-PI3K-AKT activation and decreases lymphatic sprouting (Nakayama et al., 2015; Uchida et al., 2015). Similarly, increased expression of membrane scaffolding protein caveolin-1 inhibits VEGFR3 activation by attenuating VEGFR3 autophosphorylation and ERK1/2 activation. (Galvagni et al., 2007) (Figure 1B).

#### Modulation of VEGFR3 activation by the extracellular matrix (ECM)

The composition of the ECM exerts significant influence on LEC function by modulating VEGFR3 activation. For example, the matrix proteoglycan heparan sulfate (HS) interacts with VEGFC via the syndecan-4 proteoglycan co-receptor on LECs to promote VEGFR3-mediated activation of ERK1/2 (Yin et al., 2011; Johns et al., 2016). The carbohydrate-binding protein C-type lectin domain family 14 member A (CLEC14A) receptor increases cellular responsiveness to VEGFC by increasing cellular VEGFR3 protein expression (Lee et al., 2017). The carbohydrate–binding protein galectin-8 is also thought to promote VEGFC-mediated lymphangiogenesis by coordinating crosstalk at the plasma membrane between podoplanin, integrins, and VEGFC/VEGFR3 (SIMONS et al., 2016). Matrix metalloproteinase protein (MMP) ADAMTS3, in cooperation with CCBE1, is essential for proteolytic activation of mature VEGFC (Jeltsch et al., 2014; Jha et al., 2017). Initial studies by Bos et al. revealed that CCBE1 deletion alone did not alter VEGFR3 activation in mice, but subsequent studies have revealed that CCBE1 and ADAMTS3 can greatly increase the efficiency of lymphangiogenesis via promoting activation of mature forms of VEGFC (Bos et al., 2011). Other MMPs, such as MMP-2 and MMP-9, directly and indirectly facilitate LEC tube formation and lymphangiogenesis by modulating VEGFC/VEGFR3 signaling *via* incompletely understood mechanisms (Bruyère et al., 2008; Du et al., 2017). Lysyl oxidase-like protein 2 (LOXL2), an enzymatic regulator of matrix composition, promotes lymphangiogenesis by stimulating downstream AKT and ERK phosphorylation, at least partially *via* VEGFR3 and Snail-dependent mechanisms (Wang et al., 2019; Wang et al., 2017).

#### Modulation of VEGFR3 signaling by mechanical forces

*Integrins* are the proteins found on cells that help attach cells to other cells and extracellular matrix (ECM). Thus, integrins and help cells receive signals from surrounding environment and control expression of gene activity (TAKADA et al., 2007). Changes in mechanical forces and interactions between matrix fibronectin and transmembrane integrin receptor α _5_ β _1_ increase VEGFR3 kinase transactivation and PI3K-AKT signaling (ZHANG et al., 2005). Integrin-mediated VEGFR3 transactivation depends on the recruitment of c-Src, a non-receptor–associated tyrosine kinase (Galvagni et al., 2010). A number of endothelial cell integrins are implicated in the regulation of angiogenesis by modulation of VEGFRs and integrin α_9_β_1_ is implicated in embryonic lymphatic development (Jin and Varner, 2004; Huang et al., 2000). However, nothing is known about how and which integrins regulate pathological lymphangiogenesis. Findings that α _4_ β _1_, but not α _5_ β _1_, $\alpha_{v\beta }$ _3_, or α _v_ β _5_, is essential for mediating LEC adhesion and migration through cellular fibronectin suggest defined roles for distinct integrin isotypes (Garmy-Susini et al., 2010). Experimental knockout of the integrin β _1_ subunit leads to reduced ERK phosphorylation following VEGFR3 activation, highlighting the importance of distinct integrin protein domains in regulating lymphangiogenic activity (Kumaravel et al., 2020). Periostin, a secreted ECM protein, binds α _V_ β _3_ integrins and induces lymphatic AKT signaling by promoting Src–induced VEGFR2/VEGFR3 transactivation (Kudo et al., 2012). Periostin–treated LECs have increased formation of focal adhesions and increased migration (Kudo et al., 2012). Integrin-linked kinase (ILK) constitutively dampens integrin activity by physically blocking VEGFR3 and β _1_ integrin interactions until threshold levels of mechanical stimulation are met to prevent excessive lymphangiogenesis, illustrating tightly controlled coordination of LEC responses to environmental cues (Urner et al., 2019). Within the cytoplasm, ADP ribosylation factor 6 (Arf6), a small GTPase, attenuates β _1_ integrin internalization and matrix-directed lymphangiogenesis (Lin et al., 2017).

*Notch signaling* has been implicated in modulating lymphangiogenic activities in LECs, through VEGFR3 regulation causing both positive and negative effects on lymphatic sprouting, suggesting highly context-dependent regulatory functions (Niessen et al., 2011; Zheng et al., 2011; Fatima et al., 2014; Geng et al., 2020; Choi et al., 2017b; Min et al., 2016). Mechanistically, Niessen et al., proposed inhibition of Notch1-Dll4 signaling attenuates EphrinB2 expression, thereby altering VEGFR3 signal transduction (Niessen et al., 2011). In contrast, Zheng *et al* reported attenuation of LEC responsiveness to VEGF-A stimulation (but not VEGFC) most likely via downregulated VEGF/VEGFR2 pathway-associated downstream signaling (Zheng et al., 2011). Importantly, Dll4-Notch1 activity did not appear to alter VEGFR2 phosphorylation, but rather exerted its effects by decreasing downstream signaling by an unknown related secondary messenger (Zheng et al., 2011). Later on, Murtomaki et al. demonstrated Notch1 modulates LEC responsiveness to VEGFC stimulation by attenuating ERK activation (Niessen et al., 2011; Shawber et al., 2007; Muley et al., 2022). Following translocation of its proteolytically cleaved intracellular domain into the nucleus, Notch1 mediates recruitment of the CBF-1/Suppressor of Hairless/Lag1 (CSL) transcription factor complex to increase VEGFR3 transcription and decrease VEGFR2 mRNA synthesis in LECs (Shawber et al., 2007; Muley et al., 2022). Subsequently, Bernier-Latmani *et al* reported VEGFR3-driven Dll4 expression was necessary for LEC survival and sprouting activity (Bernier-Latmani et al., 2015). Laminar shear stress (LSS) stimulation of mechanosensitive ion channel protein PIEZO1 also inhibits Notch signaling in LECs, which results in increased lymphatic sprouting activity (Choi et al., 2017b).

*Shear stress* induced by flow stimulates PIEZO1 mechanosensors that activates the transmembrane ion channel calcium release–activated calcium channel protein 1 (ORAI1), which in turn increases VEGFC gene transcription by recruiting transcription factors Kruppel-like factors 2 and 4 (Choi et al., 2022; Choi et al., 2017a).Indeed one study indicated that LECs migrated and formed vessels in the location of preformed fluid channels, suggesting that fluid flow precedes vessel formation (Boardman and Swartz, 2003; Planas-Paz et al., 2012; Fontana et al., 2020). Increased interstitial fluid pressure was associated with increased rates of peritumor lymphangiogenesis and metastases (ROFSTAD et al., 2014). Laminar shear stress-mediated activation of surface CD31 and subsequent recruitment of adherens junction protein VE-cadherin promotes phosphorylation of VEGFR2 and VEGFR3 via VEGF ligand–independent mechanisms (Coon et al., 2015). VE-cadherin transmembrane domains also physically interact with VEGFR3 to promote integrin co-activation and downstream PI3K-AKT axis signaling (Coon et al., 2015; Chen and Tzima, 2009). Additionally, VE-cadherin is required for VEGFR3 surface presentation in LECs, thereby regulating endothelial responsiveness to VEGFC stimulation (Harris et al., 2022). In mature lymphatic valves, VE-cadherin requires an intact AKT signaling node to mediate continuous expression of the transcription factor FOXC2 to prevent lymphatic valvular regression (Yang et al., 2019).

Environmental stimuli also affect VEGFR3 expression and signaling. For example, changes in matrix stiffness result in activation of the transcription factor GATA2, which directly increases VEGFR3 expression by binding intron 1 of the VEGFR3 gene (Frye et al., 2018). Similarly, flow shear stress increases VEGFR3 transcription in uterine microvascular endothelial cells via incompletely understood mechanisms (Park et al., 2017). Wnt secretion from LECs in response to shear stress is regulated by PROX-1, leading to the expression of transcription factor forkhead box protein C2 (FOXC2) and GATA2 (Cha et al., 2018). In experimental hypertensive heart failure models, chronic pressure overload leads to reduced VEGFR3 and VEGFC mRNA transcription within cardiac lymphatics (Lin et al., 2021).

### Cellular regulation of VEGFR3 expression and processing

**FIGURE 2:**
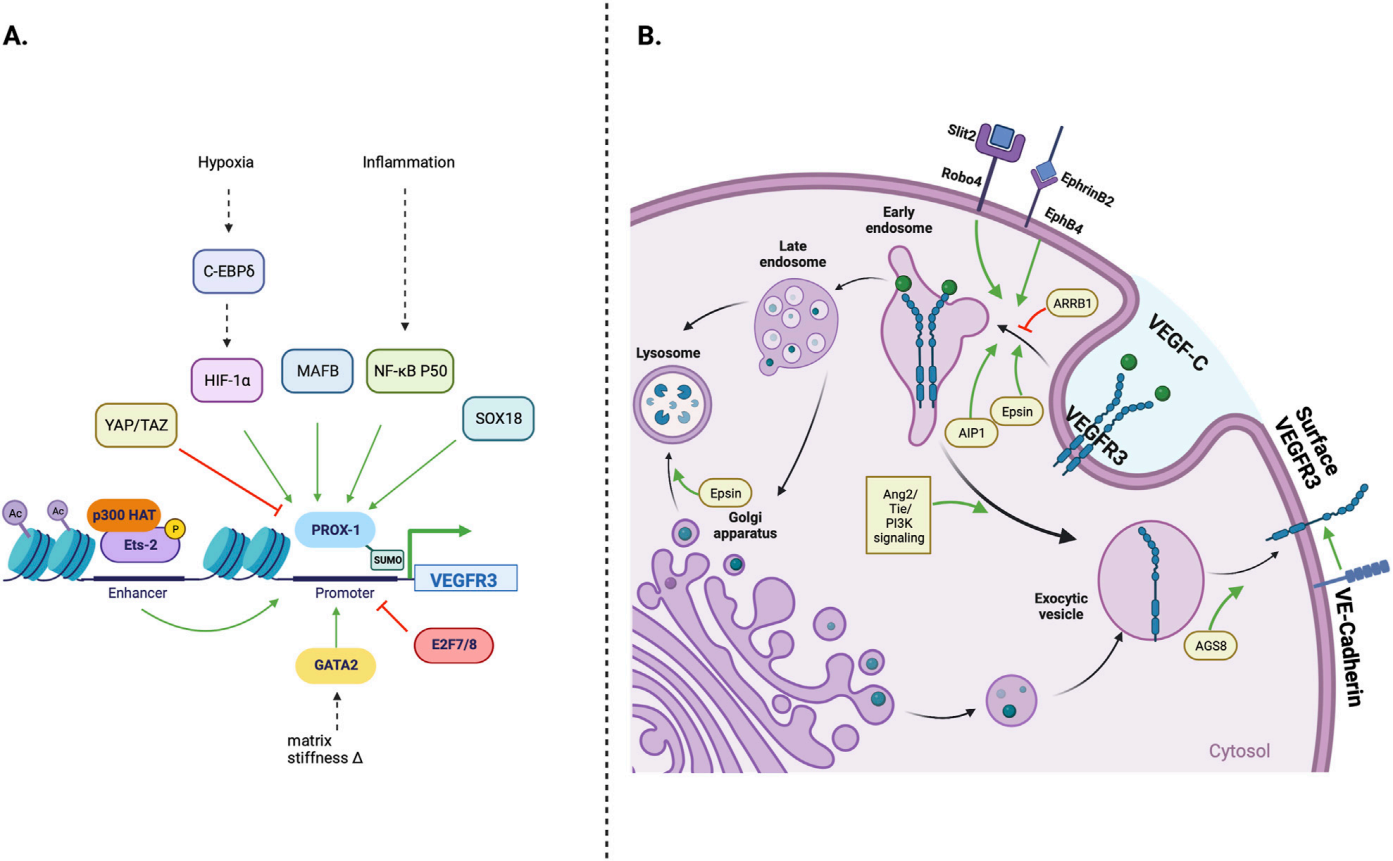
Cellular regulation of VEGFR3 expression; **(A)** The FLT4 (VEGFR3) gene promoter is primarily under Prox-1 transcription factor control. Environmental stressors such as hypoxia and inflammation can stimulate lymphangiogenesis by increasing Prox-1 activation. MAFB and SOX18 transcription factors, which play important roles in early lymphatic development, can also directly increase Prox-1 activity, while Yap/Taz transcription factors decrease Prox-1 activity and downstream VEGFR3 transcription **(B)** Transmembrane and intracellular proteins control VEGFR3 trafficking within lymphatic endothelial cells, which alters lymphangiogenic stimulatory capacity. Robo4 and EphB4 receptors promote VEGFR3 internalization, while VE-Cadherin maintains surface VEGFR3 availability. Cytoplasmic adapter proteins Epsin 1/2 and AIP1 promote VEGFR3 internalization/degradation, while ARRB1 and AGS8 promote surface VEGFR3 availability.

Lymphangiogenesis is also modulated by regulating VEGFR3 expression and processing. This control within the cell may occur at the level of VEGFR3 transcription and post-translational modification, or cell membrane receptor expression, which are summarized in Figure 2. Transcriptional Regulation of VEGFR3 expression occurs at many levels and is modulated by transcription factors, hypoxic environment, and epigenetic modifications of the regulatory regions of the VEGFR3 gene. In addition to transcriptional regulation VEGFR3 is also regulated at post-translational level as described below.

#### Transcription factors

Many transcription factors regulate VEGFR3 expression. PROX-1, the most notable, is necessary for LEC differentiation during development and maintenance of postnatal lymphatic phenotype (Johnson et al., 2008). PROX-1 dose-dependently increases LEC VEGFR3 transcription by binding promoter regions of VEGFR3 gene (Srinivasan et al., 2014). The expression and function of PROX-1 itself is also regulated by other transcription factors and signaling molecules. For example, MafB increases PROX-1 expression in developing lymphatics and pathologic tumor lymphangiogenesis and is important in the fine patterning of lymphatics and smooth muscle coverage of developing lymphatic networks (Dieterich et al., 2015; Dieterich et al., 2020; Rondon-Galeano et al., 2020). During development, Sox18–binding of the promoter region increases the expression of PROX-1 (Cermenati et al., 2013; François et al., 2008). Transcription factor NF- κ B also increases PROX-1 and VEGFR3 expression in the lymphatic vessels of some organs (Flister et al., 2010). This regulatory interaction is noteworthy since it allows inflammatory signaling pathways, such as the tumor necrosis factor superfamily-15 (TNFS15)/death receptor 3 (DR3) axis, to increase VEGFR3 transcription via NF- κ B activation (Qin et al., 2015; Zhang et al., 2019).

VEGFC-VEGFR3 signaling activates a positive feedback loop that increases the expression of VEFGR3 and PROX-1 (Srinivasan et al., 2014). The mechanisms that regulate this positive feedback loop are incompletely understood. However, VEGFR3 stimulation selectively increases the expression of transcription factors, including MafB and Sox-18, that directly regulate Prox-1 expression (Dieterich et al., 2015). Additionally, post-translational modification of PROX-1 by Small ubiquitin-like modifier 1 (SUMO-1) enhances its capacity to increase VEGFR3 mRNA transcription and increases LEC proliferation, sprouting, and tube formation (Pan et al., 2009).

The expression of PROX-1 (and VEGFR3) is also negatively controlled by transcription factors that are activated in the developing embryo or in response to inflammation. For example, hyperactivation of Hippo pathway effectors YAP and TAZ during embryogenesis decreases PROX-1 expression and impairs lymphatic development (Cho et al., 2019). Similarly, activation of these transcription factors in adult mice decreases lymphangiogenesis in corneal lymphangiogenesis assays (Cho et al., 2019). Atypical E2f transcription factors 7 and 8 also decrease VEGFR3 transcription during developmental lymphangiogenesis by suppressing VEGFR3 gene promoter activity (Weijts et al., 2013).

*Tbx1*, which encodes a T box transcription factor involved in DiGeorge syndrome, activated VEGFR3 transcription via enhancer binding. In the absence of this gene, VEGFR3 expression levels in LECs are not sustained, leading to a failure of lymphatic vessel maintenance (Chen et al., 2010). Similarly, Etv2, an ETS transcription factor, is necessary for lymphangiogenesis as seen in zebrafish through its direct upregulation of *flt4* expression (Davis et al., 2018).

#### Hypoxic environments

Although the role of hypoxia in the regulation of blood endothelial cell VEGFR signaling has been studied extensively, little is known about its effect on VEGFR3 signaling in LECs. In cultured human lung–derived LECs, hypoxia increases expression of transcription factor C/EBP- δ , which in turn increases expression of hypoxia–inducible factor-1 alpha (HIF-1a), VEGFC, and VEGFR3 mRNAs (Min et al., 2011; Han et al., 2019; Zampell et al., 2012). In cultured human umbilical vein endothelial cells, HIF-1a and HIF2a bind the hypoxia response element (HRE) sequence at the Prox-1 promoter, leading to increased Prox-1 transcription (Zhou et al., 2013). Blocking HIF-1a activity prevents C/EBP- δ *–*mediated increased expression of VEGFR3 and VEGFC in cultured human LECs (Min et al., 2011). C/EBP- δ knockout mice have decreased lymphatic (but not tumor cell or bone marrow cell) expression of VEGFR3 and VEGFC, increased LEC apoptosis, and decreased lymphangiogenesis in response to lung tumors (Min et al., 2011). The expression of C/EBP- δ is also regulated by inflammatory cytokines such as interleukin-1 β , IL-6, and tumor necrosis factor alpha, suggesting that this transcription factor may also play a role in inflammatory lymphangiogenesis (Takata et al., 2002).

#### Epigenetic modification

Regulatory regions around the VEGFR3 gene exhibit functional susceptibility to epigenetic modification (Hertel et al., 2014; Quentmeier et al., 2012; Yoo et al., 2020; Koltowska et al., 2021; Gauvrit et al., 2018). Activation of transcription factor Ets-2 by Ras/MAPK cascade signaling promotes p300 histone acetyltransferase–mediated acetylation of VEGFR3 gene regulatory domains to enhance transcription (ICHISE et al., 2012). Ets-2 also physically interacts with PROX-1 to synergistically stimulate the expression of VEGFR3 (Yoshida et al., 2015; Hong and Detmar, 2003). Experimental inhibition of histone deacetylase activity promotes sustained histone acetylation and increased Sp1/Sp3 transcription factor–mediated VEGFR3 expression, while reciprocal blockade of histone methylation also increases VEGFR3 gene transcription (Hertel et al., 2014). Clinically, VEGFR3 gene hypomethylation has been identified in early gastric cancer tissues and is correlated with lymph node metastasis (LI et al., 2020).

Metabolic pathways involved in cellular energy homeostasis also exert epigenetic control of VEGFR3 transcription. For example, fatty acid β -oxidation product acetyl-CoA increases the activity of p300 histone acetyltransferase in LECs, thus interacting with PROX-1 (Wong et al., 2017). Carnitine palmitoyltransferase 1A (CPT1A), the enzyme responsible for acetyl-CoA synthesis during fatty acid β -oxidation, is under the transcriptional control of PROX-1, highlighting reciprocal and complex regulatory interactions between these pathways (Wong et al., 2017). Mitochondrial respiration–associated electron transport chain (ETC.) activity also promotes VEGFR3 and PROX-1 gene expression *in vitro* and during *in vivo* lymphatic development (Ma et al., 2021). This control results from H3K4me3 and H3K27ac histone modifications in the promoter regions of PROX-1 and VEGFR3 (Ma et al., 2021). Recently, dysfunctional lipid droplet autophagy was shown to attenuate PROX-1 and VEGFR3 expression in LECs by impeding fatty acid delivery to the mitochondria (Meçe et al., 2022). Likewise, LEC exposure to oxidized low-density lipoprotein (LDL) reduces PROX-1 expression by dysregulating fatty acid metabolism and mitochondrial function (Burchill et al., 2021). Cystathionine β -synthetase (CBS), an enzyme involved in homocysteine metabolism and H_2_S production, promotes VEGFR2 and VEGFR3 transcription in human dermal lymphatic endothelial cells through unclearly defined mechanisms (Hatami et al., 2022).

#### Post-translational regulation of VEGFR3 expression

Cell surface VEGFR3 expression is regulated by a complex series of post-translational events, including changes in receptor internalization, receptor degradation, and receptor trafficking from the cytoplasm to the plasma membrane. VEGFC stimulation is an important modulator of VEGFR3 protein expression, likely as part of an autoregulatory feedback loop. VEGFC treatment dose-dependently decreases total VEGFR3 levels in cultured LECs, and this response is, at least partially, mediated by enhanced lysosomal degradation and decreased VEGFR3 transcription (Han et al., 2014). VEGFC-mediated VEGFR3 trafficking is also modulated by other signaling pathways. For example, EphB4 receptor activation by EphrinB2 or intracellular activation of ASK1-interacting protein (AIP1) increases VEGFC–induced VEGFR3 internalization and decreases the half-life stability of internalized VEGFR3 (Wang et al., 2010; Zhou et al., 2014). Epsins, ubiquitin–binding endocytic clathrin adapter proteins, also increase VEGFR3 membrane internalization and degradation by increasing proteolytic degradation of Golgi-bound VEGFR3 (Bhattacharjee et al., 2021; Liu et al., 2014; Wu et al., 2018). Conversely, within the cytoplasm, ARRB1 counteracts VEGFC–induced VEGFR3 internalization and degradation (Ma et al., 2019).

Other signaling cascades regulate cell surface expression of VEGFR3 independent of VEGFC. For example, Slit homolog 2 protein (Slit2), a ligand of the roundabout receptors (Robo), decreases surface VEGFR3 levels by increasing receptor internalization without altering VEGFR3 transcription (Yu et al., 2014). In contrast, VE-cadherin increases the stability of cell surface VEGFR3 molecules, which is more fully explored in subsequent sections (Harris et al., 2022). Likewise, the activator of G-protein signaling 8 (AGS8)—a receptor–independent accessory protein for heterodimeric G-proteins—increases cell surface presentation of VEGFR3 possibly by increasing receptor trafficking from the cytoplasm to the plasma membrane (Sato et al., 2006; Sakima et al., 2018).

## Intracellular VEGFR3 signaling pathways

### VEGFR3-PI3K/AKT signaling

**FIGURE 3:**
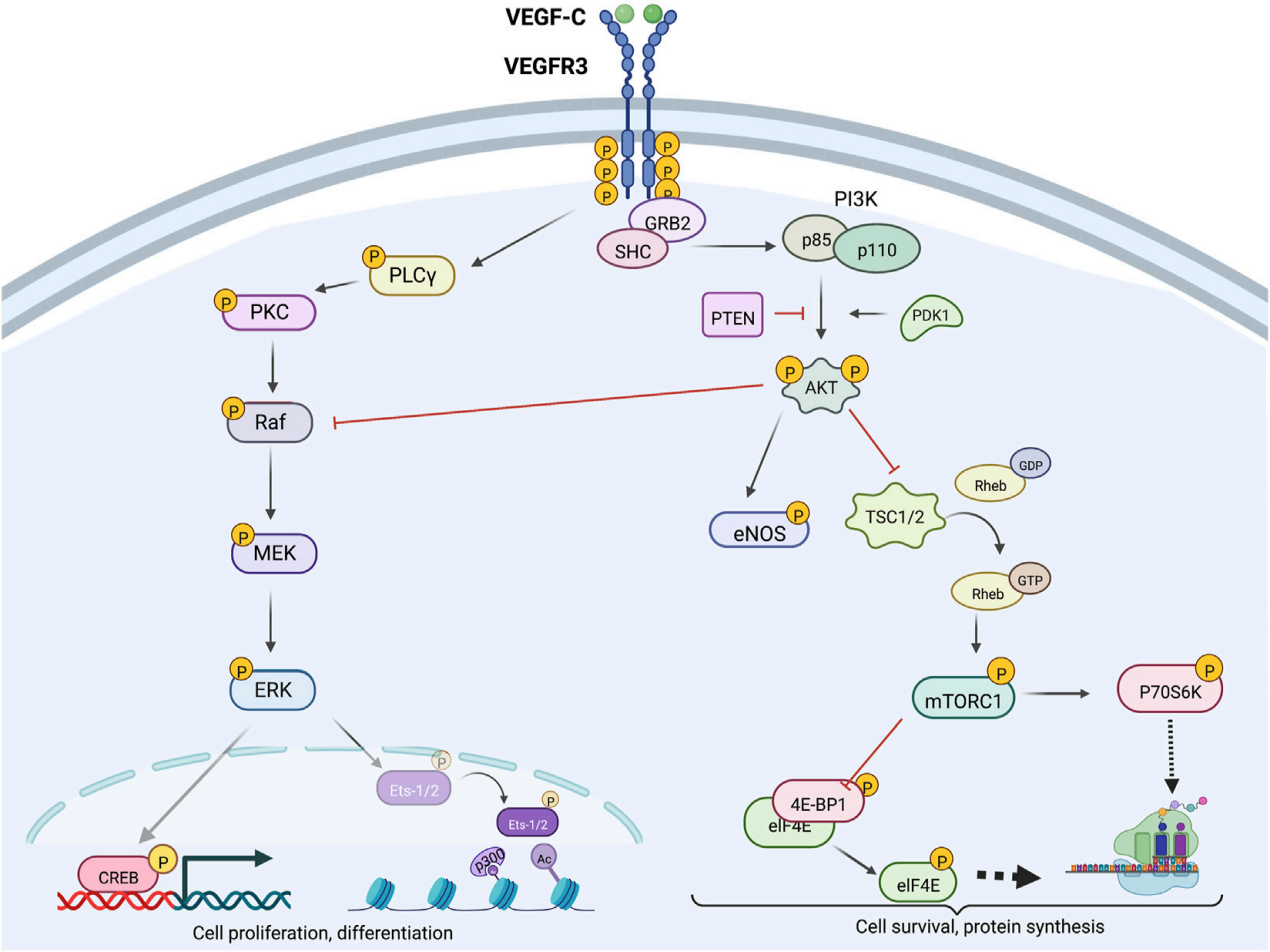
Intracellular VEGFR3 signaling; VEGFR3 signaling can be strategically enhanced or inhibited at various levels via targeting of molecular proteins located upstream or downstream from AKT and ERK. Downstream ERK effectors CREB and Ets-2 promote cell proliferation and differentiation, while downstream AKT effectors mTORC1 and eNOS primarily promote cell survival and growth functions. Crosstalk between the AKT and ERK signaling cascades allows context-dependent fine-tuned control of lymphatic endothelial cell function.

Activated VEGFR3 phosphorylates PI3K, which canonically converts membrane–bound phosphatidylinositol 4,5-bisphosphate (PIP_2_) to phosphatidylinositol 3,4,5-trisphosphate (PIP_3_) (PORTA et al., 2014; Martinez-Corral et al., 2020; Coso et al., 2012). PIP_3_ subsequently promotes AKT phosphorylation, thereby activating a variety of downstream mediators (e.g., mammalian target of rapamycin [mTOR], ribosomal protein S6 kinase beta-1 [P70S6K], endothelial nitric oxide synthase [eNOS], and phospholipase C, gamma 1 [PLCγ1]) that regulate LEC migration, proliferation, nitric oxide synthesis, and protection from apoptosis, as shown in Figure 3 (Coso et al., 2012; Luo et al., 2012; Hori et al., 2020). Specifically, PI3K regulatory subunits (p85 α , p55 α , p50 α ) encoded by the *Pik3r1* gene play important roles in promoting normal lymphangiogenic signaling (but not angiogenesis), as seen in experiments by Mouta-Bellum *et al* revealing insufficient dermal, diaphragmatic, and intestinal lymphatic development in *Pik3r1* knockout mice (Mouta-Bellum et al., 2009). Phosphoinositide-dependent kinase 1 (PDK1) stimulates downstream PI3K signaling by increasing AKT activation by PI3K (Harris, 2003). PDK1 activity is critical for AKT activation under normal conditions (Harris, 2003). In contrast, phosphatase and tensin homolog (PTEN), an enzyme that catalyzes dephosphorylation of PIP_3_ to PIP_2_, attenuates AKT activation by PI3K (Jiang and Liu, 2009).

Intracellular changes in PI3K/AKT signaling may potentially contribute to increased LEC proliferation, migration, and differentiation, which is important to consider in some pathologic settings. For example, LECs with *PIK3R3* and *PIK3CA* gene mutations derived from lymphatic malformation lesions exhibit PI3K overexpression, constitutive AKT hyperphosphorylation, and increased mTOR activation (Boscolo et al., 2015; Osborn et al., 2015). In one particular study, these patient-derived LECs also exhibited elevated VEGFR3 and Neuropilin-2 expression, thus making it difficult to determine if downstream secondary messenger changes primarily account for the apparent upregulation in *in vitro* lymphangiogenic capacity (Boscolo et al., 2015). In another similar *in vitro* study, however, lymphatic malformation-derived LECs were highly susceptible to treatment with the mTOR pathway inhibitor sirolimus, but not VEGFR3 blockade, initially suggesting upstream VEGFC-VEGFR3 signaling does not play a significant role in the development of hyperproliferative phenotypes in lymphatic malformations (Osborn et al., 2015). Later experiments in 2020 by Martinez-Corral *et al* suggest that elevated lymphangiogenesis seen in *PIK3CA* ^*H1047R*^-driven microcystic lymphatic malformations requires intact upstream VEGFC-VEGFR3 signaling, with maximal regression of lymphatic overgrowth phenotypes following *in vivo* treatment with both VEGFC trap and sirolimus, suggesting the need for a multi-pronged therapeutic approach in the management of these disorders (Martinez-Corral et al., 2020).

As suggested by the studies in the previous paragraph, modulation of intracellular PI3K/AKT signaling continues to have clinical implications for drug development. For example, our group recently showed that LEC–specific deletion of PTEN increases AKT phosphorylation, leading to increased lymphangiogenesis and lymphatic function in a variety of organ systems, including the skin, heart, lungs kidney, and intestines (Kataru et al., 2021). New lymphatics that were generated in LEC PTEN knockout mice, in contrast to those formed in response to recombinant VEGFC injection, were not leaky and had normal expression of tight junction proteins. These findings suggest that modulation of intracellular signaling pathways in LECs may be a viable and more targeted method to improve lymphatic function and may avoid off-target effects of supraphysiological doses of VEGFC.

### VEGFR3-Ras/MAPK signaling

During embryonic lymphatic development, the Ras signaling axis plays a critical role in promoting LEC differentiation through activation of the downstream effector p42/MAPK, otherwise known as ERK1/2. During embryologic lymphatic sprouting and differentiation, VEGFC-VEGFR3-ERK signaling promotes G_0_/G_1_ cell cycle arrest by increasing p53/p21 and p27 activity (Jerafi-Vider et al., 2021). ERK blockade decreases cellular p53 and p27 levels and attenuates endothelial sprouting activity; this effect is rescued by p53 and p21 overexpression, suggesting that the Ras/MAPK axis has a context–dependent role in regulating lymphatic mitogenesis (Jerafi-Vider et al., 2021). Notably, VEGFR3–autonomous ERK activity alone is not sufficient for the regulation of dorsal cell migration following LEC separation from the cardinal vein, suggesting that other signaling pathways activated by VEGFR3 are also needed for regulating lymphangiogenic activity (Jerafi-Vider et al., 2021).

Much less is understood about the role of Ras signaling in post-developmental lymphangiogenesis. In many cell types, G-protein–coupled receptor (GPCR)–mediated Ras activation is the major driver of ERK1/2 phosphorylation via the canonical Ras-Raf-MEK-ERK signaling cascade (TAKAHASHI et al., 1999). In contrast, in blood and lymphatic endothelial cells, vascular growth factor–induced Raf-MEK-ERK1/2 stimulation is indirectly mediated by protein kinase C (PKC) (Mäkinen et al., 2001b). Activated ERK primarily stimulates cell proliferation and differentiation in LECs, and has physiologic crosstalk with the PI3K/AKT axis (Bui and Hong, 2020; Heckman et al., 2008; Deng et al., 2013). For example, partial inhibition of PI3K-AKT signaling in cultured LECs increases ERK1/2 phosphorylation *via* release of AKT1–mediated negative feedback inhibition of Raf1 (Boscolo et al., 2015; Ren et al., 2010).

To determine the role of upstream Ras activity in post-differentiated LECs, Ichise *et al* developed a LEC-specific Ras overexpression model in mice (ICHISE et al., 2010). In cultured cells derived from these mutant mice, constitutive GTP-Ras activity promoted sustained MAPK phosphorylation and tube formation under serum deprivation conditions, while wildtype LECs failed to form tubular networks (ICHISE et al., 2010). Ras blockade reduced mutant LEC viability without altering cell apoptosis (ICHISE et al., 2010). The pro-lymphangiogenic effects of Ras hyperactivation were attenuated by VEGFR3 kinase inhibitors, supporting the hypothesis that Ras activation can induce cross-talk with the PI3K signaling cascade (ICHISE et al., 2010). Additional knockout experiments revealed that Ras stimulates transcription of VEGFR3 in a dose–dependent manner and that this response is required for Ras–mediated lymphangiogenesis (ICHISE et al., 2010). The Ras-GTPase RASA1, a negative regulator of Ras activity, plays an important role in modulating LEC responses to environmental VEGFC gradients (Lapinski et al., 2012). RASA1 deletion increases lymphatic proliferation and survival *in vitro* by increasing VEGFC activation of ERK and AKT (Lapinski et al., 2012). Consistent with this, *in vivo* lymphatic hyperplasia resulting from RASA1 knockout is reversed by anti-VEGFR3 antibody treatment (Lapinski et al., 2012).

Downstream ERK1/2 phosphorylation targets include cAMP response element (CRE)–binding protein (CREB), a transcription factor that increases expression of cell survival–promoting genes, and nuclear transcription factors Ets-1 and Ets-2 that regulate VEGFR3 mRNA and protein expression, as summarized in Figure 3 (Mäkinen et al., 2001b; Heckman et al., 2008; ICHISE et al., 2012). Phospho-CREB, along with molecular coactivator CREB–binding protein (CBP), interacts with regulatory CRE regions of gene promoters to increase transcriptional activity (Andrisani, 1999; Vo and Goodman, 2001). Ets-1/2 knockdown in mouse LECs also decreases VEGFR2 and PECAM1 gene expression; however, these reductions are not as significant as those noted for VEGFR3 (ICHISE et al., 2012).

### Intracellular modulation of VEGFR3 tyrosine kinase domains

Within the cytoplasm, intracellular VEGFR3 tyrosine kinase domains mediate the recruitment of molecular adapters such as growth factor receptor–bound protein 2 (Grb2) and Src homology and collagen (Shc) domain proteins to promote the activation of the PI3K-AKT and Raf-ERK1/2 signaling axes (Salameh et al., 2005; DENG et al., 2015). Multifunctional adapter protein β-arrestin 1 (ARRB1) interacts with VEGFR3 kinase domains to preferentially promote AKT activation (Ma et al., 2019). In contrast, Sprouty-related, EV1H domain-containing proteins (SPREDS) compete with Grb2 to inhibit ERK axis engagement (Taniguchi et al., 2007). Other adapter proteins such as CRKI/II, which are responsible for activating pathways such as the JNK and p38 cascades, may also relay lymphangiogenic signals (reviewed elsewhere in more detail) (YOSHIMATSU et al., 2016; Koch et al., 2011; Mäkinen et al., 2001b).

### Accessory crosstalk with VEGFR3/AKT/ERK signal transduction

Other signaling pathways also regulate lymphangiogenesis by modulating VEGFR3 activation or by interacting with shared downstream signaling cascades.

*Adrenomedullin-calcitonin-like receptor* (CLR)/RAMP2 signaling is capable of transactivating VEGFR3 via c-Src–dependent mechanisms and may also indirectly regulate VEGFC signaling in LECs by stimulating Dll4 Notch ligand expression (Geng et al., 2020; Harris et al., 2022). In inflammatory settings, COX-2 and prostaglandin E2 (PGE2) stimulate the receptor EP4 to increase lymphangiogenesis (Nandi et al., 2017; Lyons et al., 2014). EP4 receptor activation directly promotes lymphatic AKT and ERK phosphorylation via a non-canonical mechanism and indirectly stimulates VEGFR3 signaling by increasing VEGF-D expression (Nandi et al., 2017).

*Fibroblast growth factor 2* (bFGF) synergistically increases LEC responsiveness of VEGFR3 to VEGFC by enhancing downstream AKT and ERK activation (Kumaravel et al., 2020; Cao et al., 2012). Other growth factors, such as insulin-like growth factor 1 (IGF-1) and hepatocyte growth factor (HGF), also activate AKT and ERK with resultant increased lymphangiogenesis; however, these responses are independent of VEGFR3 (Björndahl et al., 2005; Cao et al., 2006; Kajiya et al., 2005).

*Endothelin receptor B*, (a GPCR) in LECs, when stimulated by endothelin-1 promotes Src–dependent VEGFR3 transactivation and synergistically increases AKT and MAPK signaling in response to VEGFC administration (Spinella et al., 2009; Spinella et al., 2013). In contrast, sphingosine-1-phosphate receptor 1 (S1PR1), another GPCR, dampens LEC responsiveness to VEGFC stimulation under LSS culture conditions but not static culture conditions, primarily via β-arrestin pathway signaling (Geng et al., 2020). S1PR1 deletion results in lymphatic hyper sprouting and aberrant vessel morphogenesis during embryonic lymphatic development (Geng et al., 2020). Endostatin serves as a negative regulator of lymphangiogenesis in LECs by stimulating cell surface nucleolin, which attenuates ERK1/2 activation resulting from VEGFC stimulation (Zhuo et al., 2010).

Activation of VEGFR3 in LECs is coordinated by a variety of mechanisms, including regulation by co-receptors, modulation by the ECM and mechanical forces, regulation of VEGFR3 mRNA expression and post-translational modification, cell surface expression, and intracellular signaling pathways. Downstream stimulation of the PI3K-AKT and Ras-MAPK signaling axes directly or indirectly activates interdependent pathways that regulate cellular proliferation, survival, migration, responsiveness to lymphangiogenic cytokines, and tubule formation. For future directions it will be interesting to investigate how VEGFR3 signaling pathway is regulated in different organs systems of mammalian body. For example, adult intestine and meningeal lymphatics needs constant VEGFR3/VEGFC signaling for maintenance but not skin lymphatics (Nurmi et al., 2015; Antila et al., 2017). Additionally, considering VEGFR3 signaling is critical for sprouting angiogenesis and blood vascular growth it will be interesting to learn how signaling pathways are activated in vascular endothelial cells compared to LECs (Tammela et al., 2008). In addition, aspects related to VEGFR3 internalization, localization and overall availability still needs to be thoroughly studied. Further characterization and understanding of these signaling interactions will allow researchers to design targeted interventions that can either increase or decrease lymphangiogenesis with limited off-target effects, depending on the clinical scenario.

## References

[B1] Alam A. Herault J. P. Barron P. Favier B. Fons P. Delesque-Touchard N. (2004). Heterodimerization with vascular endothelial growth factor receptor-2 (VEGFR-2) is necessary for VEGFR-3 activity. Biochem. Biophys. Res. Commun. 324, 909–915. 10.1016/j.bbrc.2004.08.237 15474514

[B2] Andrisani O. M. (1999). CREB-mediated transcriptional control. Crit. Rev. Eukaryot. Gene Expr. 9, 19–32. 10.1615/critreveukaryotgeneexpr.v9.i1.20 10200909

[B3] Antila S. Karaman S. Nurmi H. Airavaara M. Voutilainen M. H. Mathivet T. (2017). Development and plasticity of meningeal lymphatic vessels. J. Exp. Med. 214, 3645–3667. 10.1084/jem.20170391 29141865 PMC5716035

[B4] Aprelikova O. Pajusola K. Partanen J. Armstrong E. Alitalo R. Bailey S. K. (1992). FLT4, a novel class III receptor tyrosine kinase in chromosome 5q33-qter. Cancer Res. 52, 746–748. 1310071

[B5] Bernier-Latmani J. Cisarovsky C. Demir C. S. Bruand M. Jaquet M. Davanture S. (2015). DLL4 promotes continuous adult intestinal lacteal regeneration and dietary fat transport. J. Clin. Invest. 125, 4572–4586. 10.1172/JCI82045 26529256 PMC4665794

[B6] Bhattacharjee S. Lee Y. Zhu B. Wu H. Chen Y. Chen H. (2021). Epsins in vascular development, function and disease. Cell Mol. Life Sci. 78, 833–842. 10.1007/s00018-020-03642-4 32930806 PMC7902377

[B7] Björndahl M. Cao R. Nissen L. J. Clasper S. Johnson L. A. Xue Y. (2005). Insulin-like growth factors 1 and 2 induce lymphangiogenesis *in vivo* . Proc. Natl. Acad. Sci. U. S. A. 102, 15593–15598. 10.1073/pnas.0507865102 16230630 PMC1266150

[B8] Boardman K. C. Swartz M. A. (2003). Interstitial flow as a guide for lymphangiogenesis. Circulation Res. 92, 801–808. 10.1161/01.RES.0000065621.69843.49 12623882

[B9] Borg J. P. Delapeyrière O. Noguchi T. Rottapel R. Dubreuil P. Birnbaum D. (1995). Biochemical characterization of two isoforms of FLT4, a VEGF receptor-related tyrosine kinase. Oncogene 10, 973–984. 7898938

[B10] Bos F. L. Caunt M. Peterson-Maduro J. Planas-Paz L. Kowalski J. Karpanen T. (2011). CCBE1 is essential for mammalian lymphatic vascular development and enhances the lymphangiogenic effect of vascular endothelial growth factor-C *in vivo* . Circ. Res. 109, 486–491. 10.1161/CIRCRESAHA.111.250738 21778431

[B11] Boscolo E. Coma S. Luks V. L. Greene A. K. Klagsbrun M. Warman M. L. (2015). AKT hyper-phosphorylation associated with PI3K mutations in lymphatic endothelial cells from a patient with lymphatic malformation. Angiogenesis 18, 151–162. 10.1007/s10456-014-9453-2 25424831 PMC4366356

[B12] Bruyère F. Melen-Lamalle L. Blacher S. Roland G. Thiry M. Moons L. (2008). Modeling lymphangiogenesis in a three-dimensional culture system. Nat. Methods 5, 431–437. 10.1038/nmeth.1205 18425139

[B13] Bui H. M. Enis D. Robciuc M. R. Nurmi H. J. Cohen J. Chen M. (2016). Proteolytic activation defines distinct lymphangiogenic mechanisms for VEGFC and VEGFD. J. Clin. Invest. 126, 2167–2180. 10.1172/JCI83967 27159393 PMC4887177

[B14] Bui K. Hong Y. K. (2020). Ras pathways on Prox1 and lymphangiogenesis: insights for therapeutics. Front. Cardiovasc Med. 7, 597374. 10.3389/fcvm.2020.597374 33263009 PMC7688453

[B15] Burchill M. A. Finlon J. M. Goldberg A. R. Gillen A. E. Dahms P. A. Mcmahan R. H. (2021). Oxidized low-density lipoprotein drives dysfunction of the liver lymphatic system. Cell Mol. Gastroenterol. Hepatol. 11, 573–595. 10.1016/j.jcmgh.2020.09.007 32961356 PMC7803659

[B16] Cao R. Björndahl M. A. Gallego M. I. Chen S. Religa P. Hansen A. J. (2006). Hepatocyte growth factor is a lymphangiogenic factor with an indirect mechanism of action. Blood 107, 3531–3536. 10.1182/blood-2005-06-2538 16424394

[B17] Cao R. Ji H. Feng N. Zhang Y. Yang X. Andersson P. (2012). Collaborative interplay between FGF-2 and VEGF-C promotes lymphangiogenesis and metastasis. Proc. Natl. Acad. Sci. U. S. A. 109, 15894–15899. 10.1073/pnas.1208324109 22967508 PMC3465417

[B18] Cermenati S. Moleri S. Neyt C. Bresciani E. Carra S. Grassini D. R. (2013). Sox18 genetically interacts with VegfC to regulate lymphangiogenesis in zebrafish. Arterioscler. Thromb. Vasc. Biol. 33, 1238–1247. 10.1161/ATVBAHA.112.300254 23520166

[B19] Cha B. Geng X. Mahamud M. R. Zhang J. Y. Chen L. Kim W. (2018). Complementary Wnt sources regulate lymphatic vascular development via PROX1-dependent wnt/β-catenin signaling. Cell Rep. 25, 571–584. 10.1016/j.celrep.2018.09.049 30332639 PMC6264919

[B20] Chen L. Mupo A. Huynh T. Cioffi S. Woods M. Jin C. (2010). Tbx1 regulates*Vegfr3*and is required for lymphatic vessel development. J. Cell Biol. 189, 417–424. 10.1083/jcb.200912037 20439995 PMC2867300

[B21] Chen Z. Tzima E. (2009). PECAM-1 is necessary for flow-induced vascular remodeling. Arterioscler. Thromb. Vasc. Biol. 29, 1067–1073. 10.1161/ATVBAHA.109.186692 19390054 PMC2723862

[B22] Cho H. Kim J. Ahn J. H. Hong Y. K. Mäkinen T. Lim D. S. (2019). YAP and TAZ negatively regulate Prox1 during developmental and pathologic lymphangiogenesis. Circ. Res. 124, 225–242. 10.1161/CIRCRESAHA.118.313707 30582452

[B23] Choi D. Park E. Jung E. Seong Y. J. Hong M. Lee S. (2017a). ORAI1 activates proliferation of lymphatic endothelial cells in response to laminar flow through Krüppel-like factors 2 and 4. Circ. Res. 120, 1426–1439. 10.1161/CIRCRESAHA.116.309548 28167653 PMC6300148

[B24] Choi D. Park E. Jung E. Seong Y. J. Yoo J. Lee E. (2017b). Laminar flow downregulates Notch activity to promote lymphatic sprouting. J. Clin. Invest. 127, 1225–1240. 10.1172/JCI87442 28263185 PMC5373895

[B25] Choi D. Park E. Yu R. P. Cooper M. N. Cho I. T. Choi J. (2022). Piezo1-Regulated mechanotransduction controls flow-activated lymphatic expansion. Circ. Res. 131, e2–e21. 10.1161/CIRCRESAHA.121.320565 35701867 PMC9308715

[B26] Coon B. G. Baeyens N. Han J. Budatha M. Ross T. D. Fang J. S. (2015). Intramembrane binding of VE-cadherin to VEGFR2 and VEGFR3 assembles the endothelial mechanosensory complex. J. Cell Biol. 208, 975–986. 10.1083/jcb.201408103 25800053 PMC4384728

[B27] Coso S. Zeng Y. Opeskin K. Williams E. D. (2012). Vascular endothelial growth factor receptor-3 directly interacts with phosphatidylinositol 3-kinase to regulate lymphangiogenesis. PLoS One 7, e39558. 10.1371/journal.pone.0039558 22745786 PMC3382126

[B28] Davis J. A. Koenig A. L. Lubert A. Chestnut B. Liu F. Palencia Desai S. (2018). ETS transcription factor Etsrp/Etv2 is required for lymphangiogenesis and directly regulates vegfr3/flt4 expression. Dev. Biol. 440, 40–52. 10.1016/j.ydbio.2018.05.003 29753018 PMC6054491

[B29] Deng Y. Atri D. Eichmann A. Simons M. (2013). Endothelial ERK signaling controls lymphatic fate specification. J. Clin. Invest. 123, 1202–1215. 10.1172/JCI63034 23391722 PMC3582116

[B30] Deng Y. Zhang X. Simons M. (2015). Molecular controls of lymphatic VEGFR3 signaling. Arterioscler. Thromb. Vasc. Biol. 35, 421–429. 10.1161/ATVBAHA.114.304881 25524775 PMC4304921

[B31] Dieterich L. C. Klein S. Mathelier A. Sliwa-Primorac A. Ma Q. Hong Y. K. (2015). DeepCAGE transcriptomics reveal an important role of the transcription factor MAFB in the lymphatic endothelium. Cell Rep. 13, 1493–1504. 10.1016/j.celrep.2015.10.002 26549461

[B32] Dieterich L. C. Tacconi C. Menzi F. Proulx S. T. Kapaklikaya K. Hamada M. (2020). Lymphatic MAFB regulates vascular patterning during developmental and pathological lymphangiogenesis. Angiogenesis 23, 411–423. 10.1007/s10456-020-09721-1 32307629 PMC7311381

[B33] Dixelius J. Makinen T. Wirzenius M. Karkkainen M. J. Wernstedt C. Alitalo K. (2003). Ligand-induced vascular endothelial growth factor receptor-3 (VEGFR-3) heterodimerization with VEGFR-2 in primary lymphatic endothelial cells regulates tyrosine phosphorylation sites. J. Biol. Chem. 278, 40973–40979. 10.1074/jbc.M304499200 12881528

[B34] DU H. T. DU L. L. Tang X. L. Ge H. Y. Liu P. (2017). Blockade of MMP-2 and MMP-9 inhibits corneal lymphangiogenesis. Graefes Arch. Clin. Exp. Ophthalmol. 255, 1573–1579. 10.1007/s00417-017-3651-8 28669039

[B35] Durré T. Morfoisse F. Erpicum C. Ebroin M. Blacher S. García-Caballero M. (2018). uPARAP/Endo180 receptor is a gatekeeper of VEGFR-2/VEGFR-3 heterodimerisation during pathological lymphangiogenesis. Nat. Commun. 9, 5178. 10.1038/s41467-018-07514-1 30518756 PMC6281649

[B36] Fatima A. Culver A. Culver F. Liu T. Dietz W. H. Thomson B. R. (2014). Murine Notch1 is required for lymphatic vascular morphogenesis during development. Dev. Dyn. 243, 957–964. 10.1002/dvdy.24129 24659232 PMC4062592

[B37] Flister M. J. Wilber A. Hall K. L. Iwata C. Miyazono K. Nisato R. E. (2010). Inflammation induces lymphangiogenesis through up-regulation of VEGFR-3 mediated by NF-kappaB and Prox1. Blood 115, 418–429. 10.1182/blood-2008-12-196840 19901262 PMC2808162

[B38] Fontana F. Haack T. Reichenbach M. Knaus P. Puceat M. Abdelilah-Seyfried S. (2020). Antagonistic activities of vegfr3/flt4 and Notch1b fine-tune mechanosensitive signaling during zebrafish cardiac valvulogenesis. Cell Rep. 32, 107883. 10.1016/j.celrep.2020.107883 32668254

[B39] Fournier E. Blaikie P. Rosnet O. Margolis B. Birnbaum D. Borg J. P. (1999). Role of tyrosine residues and protein interaction domains of SHC adaptor in VEGF receptor 3 signaling. Oncogene 18, 507–514. 10.1038/sj.onc.1202315 9927207

[B40] Fournier E. Rosnet O. Marchetto S. Turck C. W. Rottapel R. Pelicci P. G. (1996). Interaction with the phosphotyrosine binding domain/phosphotyrosine interacting domain of SHC is required for the transforming activity of the FLT4/VEGFR3 receptor tyrosine kinase. J. Biol. Chem. 271, 12956–12963. 10.1074/jbc.271.22.12956 8662748

[B41] François M. Caprini A. Hosking B. Orsenigo F. Wilhelm D. Browne C. (2008). Sox18 induces development of the lymphatic vasculature in mice. Nature 456, 643–647. 10.1038/nature07391 18931657

[B42] Frye M. Taddei A. Dierkes C. Martinez-Corral I. Fielden M. Ortsäter H. (2018). Matrix stiffness controls lymphatic vessel formation through regulation of a GATA2-dependent transcriptional program. Nat. Commun. 9, 1511. 10.1038/s41467-018-03959-6 29666442 PMC5904183

[B43] Galland F. Karamysheva A. Pebusque M. J. Borg J. P. Rottapel R. Dubreuil P. (1993). The FLT4 gene encodes a transmembrane tyrosine kinase related to the vascular endothelial growth factor receptor. Oncogene 8, 1233–1240. 8386825

[B44] Galvagni F. Anselmi F. Salameh A. Orlandini M. Rocchigiani M. Oliviero S. (2007). Vascular endothelial growth factor receptor-3 activity is modulated by its association with caveolin-1 on endothelial membrane. Biochemistry 46, 3998–4005. 10.1021/bi061400n 17348685

[B45] Galvagni F. Pennacchini S. Salameh A. Rocchigiani M. Neri F. Orlandini M. (2010). Endothelial cell adhesion to the extracellular matrix induces c-Src-dependent VEGFR-3 phosphorylation without the activation of the receptor intrinsic kinase activity. Circ. Res. 106, 1839–1848. 10.1161/CIRCRESAHA.109.206326 20431062

[B46] Garmy-Susini B. Avraamides C. J. Schmid M. C. Foubert P. Ellies L. G. Barnes L. (2010). Integrin alpha4beta1 signaling is required for lymphangiogenesis and tumor metastasis. Cancer Res. 70, 3042–3051. 10.1158/0008-5472.CAN-09-3761 20388801 PMC2856096

[B47] Gauvrit S. Villasenor A. Strilic B. Kitchen P. Collins M. M. Marín-Juez R. (2018). HHEX is a transcriptional regulator of the VEGFC/FLT4/PROX1 signaling axis during vascular development. Nat. Commun. 9, 2704. 10.1038/s41467-018-05039-1 30006544 PMC6045644

[B48] Geng X. Yanagida K. Akwii R. G. Choi D. Chen L. Ho Y. (2020). S1PR1 regulates the quiescence of lymphatic vessels by inhibiting laminar shear stress-dependent VEGF-C signaling. JCI Insight 5, e137652. 10.1172/jci.insight.137652 32544090 PMC7453895

[B49] Han K. Y. Chang J. H. Dugas-Ford J. Alexander J. S. Azar D. T. (2014). Involvement of lysosomal degradation in VEGF-C-induced down-regulation of VEGFR-3. FEBS Lett. 588, 4357–4363. 10.1016/j.febslet.2014.09.034 25281926 PMC4254303

[B50] Han T. Yan J. Chen H. Ji Y. Chen J. Cui J. (2019). HIF-1α contributes to tube malformation of human lymphatic endothelial cells by upregulating VEGFR-3. Int. J. Oncol. 54, 139–151. 10.3892/ijo.2018.4623 30431105 PMC6254933

[B51] Harris N. R. Nielsen N. R. Pawlak J. B. Aghajanian A. Rangarajan K. Serafin D. S. (2022). VE-cadherin is required for cardiac lymphatic maintenance and signaling. Circ. Res. 130, 5–23. 10.1161/CIRCRESAHA.121.318852 34789016 PMC8756423

[B52] Harris T. K. (2003). PDK1 and PKB/Akt: ideal targets for development of new strategies to structure-based drug design. IUBMB Life 55, 117–126. 10.1080/1521654031000115951 12822887

[B53] Hatami N. Büttner C. Bock F. Simfors S. Musial G. Reis A. (2022). Cystathionine β-synthase as novel endogenous regulator of lymphangiogenesis via modulating VEGF receptor 2 and 3. Commun. Biol. 5, 950. 10.1038/s42003-022-03923-7 36088423 PMC9464209

[B54] Heckman C. A. Holopainen T. Wirzenius M. Keskitalo S. Jeltsch M. Ylä-Herttuala S. (2008). The tyrosine kinase inhibitor cediranib blocks ligand-induced vascular endothelial growth factor receptor-3 activity and lymphangiogenesis. Cancer Res. 68, 4754–4762. 10.1158/0008-5472.CAN-07-5809 18559522

[B55] Hertel J. Hirche C. Wissmann C. Ebert M. P. Höcker M. (2014). Transcription of the vascular endothelial growth factor receptor-3 (VEGFR3) gene is regulated by the zinc finger proteins Sp1 and Sp3 and is under epigenetic control: transcription of vascular endothelial growth factor receptor 3. Cell Oncol. (Dordr) 37, 131–145. 10.1007/s13402-014-0169-5 24710631 PMC13004477

[B56] Hong Y. K. Detmar M. (2003). Prox1, master regulator of the lymphatic vasculature phenotype. Cell Tissue Res. 314, 85–92. 10.1007/s00441-003-0747-8 12883994

[B57] Hori Y. Ozeki M. Hirose K. Matsuoka K. Matsui T. Kohara M. (2020). Analysis of mTOR pathway expression in lymphatic malformation and related diseases. Pathol. Int. 70, 323–329. 10.1111/pin.12913 32067331

[B58] Huang X. Z. Wu J. F. Ferrando R. Lee J. H. Wang Y. L. Farese R. V. (2000). Fatal bilateral chylothorax in mice lacking the integrin alpha9beta1. Mol. Cell. Biol. 20, 5208–5215. 10.1128/mcb.20.14.5208-5215.2000 10866676 PMC85969

[B59] Hughes D. C. (2001). Alternative splicing of the human VEGFGR-3/FLT4 gene as a consequence of an integrated human endogenous retrovirus. J. Mol. Evol. 53, 77–79. 10.1007/s002390010195 11479678

[B60] Ichise T. Yoshida N. Ichise H. (2010). H-N- and Kras cooperatively regulate lymphatic vessel growth by modulating VEGFR3 expression in lymphatic endothelial cells in mice. Development 137, 1003–1013. 10.1242/dev.043489 20179099

[B61] Ichise T. Yoshida N. Ichise H. (2012). Ras/MAPK signaling modulates VEGFR-3 expression through Ets-mediated p300 recruitment and histone acetylation on the Vegfr3 gene in lymphatic endothelial cells. PLoS One 7, e51639. 10.1371/journal.pone.0051639 23284731 PMC3524184

[B62] Jeltsch M. Jha S. K. Tvorogov D. Anisimov A. Leppänen V. M. Holopainen T. (2014). CCBE1 enhances lymphangiogenesis via A disintegrin and metalloprotease with thrombospondin motifs-3-mediated vascular endothelial growth factor-C activation. Circulation 129, 1962–1971. 10.1161/CIRCULATIONAHA.113.002779 24552833

[B63] Jeltsch M. Karpanen T. Strandin T. Aho K. Lankinen H. Alitalo K. (2006). Vascular endothelial growth factor (VEGF)/VEGF-C mosaic molecules reveal specificity determinants and feature novel receptor binding patterns. J. Biol. Chem. 281, 12187–12195. 10.1074/jbc.M511593200 16505489

[B64] Jerafi-Vider A. Bassi I. Moshe N. Tevet Y. Hen G. Splittstoesser D. (2021). VEGFC/FLT4-induced cell-cycle arrest mediates sprouting and differentiation of venous and lymphatic endothelial cells. Cell Rep. 35, 109255. 10.1016/j.celrep.2021.109255 34133928 PMC8220256

[B65] Jha S. K. Rauniyar K. Karpanen T. Leppänen V. M. Brouillard P. Vikkula M. (2017). Efficient activation of the lymphangiogenic growth factor VEGF-C requires the C-terminal domain of VEGF-C and the N-terminal domain of CCBE1. Sci. Rep. 7, 4916. 10.1038/s41598-017-04982-1 28687807 PMC5501841

[B66] Jiang B. H. Liu L. Z. (2009). PI3K/PTEN signaling in angiogenesis and tumorigenesis. Adv. Cancer Res. 102, 19–65. 10.1016/S0065-230X(09)02002-8 19595306 PMC2933405

[B67] Jin H. Varner J. (2004). Integrins: roles in cancer development and as treatment targets. Br. J. Cancer 90, 561–565. 10.1038/sj.bjc.6601576 14760364 PMC2410157

[B68] Johns S. C. Yin X. Jeltsch M. Bishop J. R. Schuksz M. EL Ghazal R. (2016). Functional importance of a proteoglycan coreceptor in pathologic lymphangiogenesis. Circ. Res. 119, 210–221. 10.1161/CIRCRESAHA.116.308504 27225479 PMC4938725

[B69] Johnson N. C. Dillard M. E. Baluk P. Mcdonald D. M. Harvey N. L. Frase S. L. (2008). Lymphatic endothelial cell identity is reversible and its maintenance requires Prox1 activity. Genes Dev. 22, 3282–3291. 10.1101/gad.1727208 19056883 PMC2600759

[B70] Joukov V. Pajusola K. Kaipainen A. Chilov D. Lahtinen I. Kukk E. (1996). A novel vascular endothelial growth factor, VEGF-C, is a ligand for the Flt4 (VEGFR-3) and KDR (VEGFR-2) receptor tyrosine kinases. Embo J. 15, 1751–1798. 10.1002/j.1460-2075.1996.tb00521.x 8612600 PMC450088

[B71] Joukov V. Sorsa T. Kumar V. Jeltsch M. Claesson-Welsh L. Cao Y. (1997). Proteolytic processing regulates receptor specificity and activity of VEGF-C. Embo J. 16, 3898–3911. 10.1093/emboj/16.13.3898 9233800 PMC1170014

[B72] Kajiya K. Hirakawa S. Ma B. Drinnenberg I. Detmar M. (2005). Hepatocyte growth factor promotes lymphatic vessel formation and function. Embo J. 24, 2885–2895. 10.1038/sj.emboj.7600763 16052207 PMC1187946

[B73] Karkkainen M. J. Haiko P. Sainio K. Partanen J. Taipale J. Petrova T. V. (2004). Vascular endothelial growth factor C is required for sprouting of the first lymphatic vessels from embryonic veins. Nat. Immunol. 5, 74–80. 10.1038/ni1013 14634646

[B74] Kataru R. P. Baik J. E. Park H. J. Ly C. L. Shin J. Schwartz N. (2021). Lymphatic-specific intracellular modulation of receptor tyrosine kinase signaling improves lymphatic growth and function. Sci. Signal 14, eabc0836. 10.1126/scisignal.abc0836 34376570 PMC8567054

[B75] Kerber M. Reiss Y. Wickersheim A. Jugold M. Kiessling F. Heil M. (2008). Flt-1 signaling in macrophages promotes glioma growth *in vivo* . Cancer Res. 68, 7342–7351. 10.1158/0008-5472.CAN-07-6241 18794121

[B76] Koch S. Tugues S. Li X. Gualandi L. Claesson-Welsh L. (2011). Signal transduction by vascular endothelial growth factor receptors. Biochem. J. 437, 169–183. 10.1042/BJ20110301 21711246

[B77] Koltowska K. Okuda K. S. Gloger M. Rondon-Galeano M. Mason E. Xuan J. (2021). The RNA helicase Ddx21 controls Vegfc-driven developmental lymphangiogenesis by balancing endothelial cell ribosome biogenesis and p53 function. Nat. Cell Biol. 23, 1136–1147. 10.1038/s41556-021-00784-w 34750583

[B78] Korhonen E. A. Murtomäki A. Jha S. K. Anisimov A. Pink A. Zhang Y. (2022). Lymphangiogenesis requires Ang2/Tie/PI3K signaling for VEGFR3 cell-surface expression. J. Clin. Invest. 132, e155478. 10.1172/JCI155478 35763346 PMC9337826

[B79] Kudo Y. Iizuka S. Yoshida M. Nguyen P. T. Siriwardena S. B. Tsunematsu T. (2012). Periostin directly and indirectly promotes tumor lymphangiogenesis of head and neck cancer. PLoS One 7, e44488. 10.1371/journal.pone.0044488 22952986 PMC3431354

[B80] Kumaravel S. Abbey C. A. Bayless K. J. Chakraborty S. (2020). The β(1)-integrin plays a key role in LEC invasion in an optimized 3-D collagen matrix model. Am. J. Physiol. Cell Physiol. 319, C1045–c1058. 10.1152/ajpcell.00299.2020 33052069

[B81] Kuonqui K. Campbell A. C. Sarker A. Roberts A. Pollack B. L. Park H. J. (2023). Dysregulation of lymphatic endothelial VEGFR3 signaling in disease. Cells 13, 68. 10.3390/cells13010068 38201272 PMC10778007

[B82] Lapinski P. E. Kwon S. Lubeck B. A. Wilkinson J. E. Srinivasan R. S. Sevick-Muraca E. (2012). RASA1 maintains the lymphatic vasculature in a quiescent functional state in mice. J. Clin. Invest. 122, 733–747. 10.1172/JCI46116 22232212 PMC3266774

[B83] Lee S. Rho S. S. Park H. Park J. A. Kim J. Lee I. K. (2017). Carbohydrate-binding protein CLEC14A regulates VEGFR-2- and VEGFR-3-dependent signals during angiogenesis and lymphangiogenesis. J. Clin. Invest. 127, 457–471. 10.1172/JCI85145 27991863 PMC5272179

[B84] Leppänen V. M. Tvorogov D. Kisko K. Prota A. E. Jeltsch M. Anisimov A. (2013). Structural and mechanistic insights into VEGF receptor 3 ligand binding and activation. Proc. Natl. Acad. Sci. U. S. A. 110, 12960–12965. 10.1073/pnas.1301415110 23878260 PMC3740881

[B85] Li X. F. Zhang T. G. Zhang Y. X. (2020). Correlation among VEGFR3 gene promoter methylation, protein overexpression, and clinical pathology in early gastric cancer. Transl. Cancer Res. 9, 3499–3506. 10.21037/tcr.2020.03.74 35117715 PMC8798734

[B86] Lin Q. Y. Zhang Y. L. Bai J. Liu J. Q. Li H. H. (2021). VEGF-C/VEGFR-3 axis protects against pressure-overload induced cardiac dysfunction through regulation of lymphangiogenesis. Clin. Transl. Med. 11, e374. 10.1002/ctm2.374 33783987 PMC7989711

[B87] Lin Y. C. Ohbayashi N. Hongu T. Katagiri N. Funakoshi Y. Lee H. (2017). Arf6 in lymphatic endothelial cells regulates lymphangiogenesis by controlling directional cell migration. Sci. Rep. 7, 11431. 10.1038/s41598-017-11240-x 28900118 PMC5595869

[B88] Liu X. Pasula S. Song H. Tessneer K. L. Dong Y. Hahn S. (2014). Temporal and spatial regulation of epsin abundance and VEGFR3 signaling are required for lymphatic valve formation and function. Sci. Signal 7, ra97. 10.1126/scisignal.2005413 25314967 PMC4226761

[B89] Luo Y. Liu L. Rogers D. Su W. Odaka Y. Zhou H. (2012). Rapamycin inhibits lymphatic endothelial cell tube formation by downregulating vascular endothelial growth factor receptor 3 protein expression. Neoplasia 14, 228–237. 10.1593/neo.111570 22496622 PMC3323900

[B90] Lyons T. R. Borges V. F. Betts C. B. Guo Q. Kapoor P. Martinson H. A. (2014). Cyclooxygenase-2-dependent lymphangiogenesis promotes nodal metastasis of postpartum breast cancer. J. Clin. Invest. 124, 3901–3912. 10.1172/JCI73777 25133426 PMC4153700

[B91] Mäkinen T. Jussila L. Veikkola T. Karpanen T. Kettunen M. I. Pulkkanen K. J. (2001a). Inhibition of lymphangiogenesis with resulting lymphedema in transgenic mice expressing soluble VEGF receptor-3. Nat. Med. 7, 199–205. 10.1038/84651 11175851

[B92] Mäkinen T. Veikkola T. Mustjoki S. Karpanen T. Catimel B. Nice E. C. (2001b). Isolated lymphatic endothelial cells transduce growth, survival and migratory signals via the VEGF-C/D receptor VEGFR-3. Embo J. 20, 4762–4773. 10.1093/emboj/20.17.4762 11532940 PMC125596

[B93] Martinez-Corral I. Zhang Y. Petkova M. Ortsäter H. Sjöberg S. Castillo S. D. (2020). Blockade of VEGF-C signaling inhibits lymphatic malformations driven by oncogenic PIK3CA mutation. Nat. Commun. 11, 2869. 10.1038/s41467-020-16496-y 32513927 PMC7280302

[B94] Ma W. Gil H. J. Liu X. Diebold L. P. Morgan M. A. Oxendine-Burns M. J. (2021). Mitochondrial respiration controls the Prox1-Vegfr3 feedback loop during lymphatic endothelial cell fate specification and maintenance. Sci. Adv. 7, eabe7359. 10.1126/sciadv.abe7359 33931446 PMC8087398

[B95] Ma Z. Yu Y. R. Badea C. T. Kovacs J. J. Xiong X. Comhair S. (2019). Vascular endothelial growth factor receptor 3 regulates endothelial function through β-arrestin 1. Circulation 139, 1629–1642. 10.1161/CIRCULATIONAHA.118.034961 30586762 PMC6433500

[B96] Meçe O. Houbaert D. Sassano M. L. Durré T. Maes H. Schaaf M. (2022). Lipid droplet degradation by autophagy connects mitochondria metabolism to Prox1-driven expression of lymphatic genes and lymphangiogenesis. Nat. Commun. 13, 2760. 10.1038/s41467-022-30490-6 35589749 PMC9120506

[B97] Min J. H. Lee C. H. Ji Y. W. Yeo A. Noh H. Song I. (2016). Activation of dll4/notch signaling and hypoxia-inducible factor-1 alpha facilitates lymphangiogenesis in lacrimal glands in dry eye. PLoS One 11, e0147846. 10.1371/journal.pone.0147846 26828208 PMC4734677

[B98] Min Y. Ghose S. Boelte K. Li J. Yang L. Lin P. C. (2011). C/EBP-δ regulates VEGF-C autocrine signaling in lymphangiogenesis and metastasis of lung cancer through HIF-1α. Oncogene 30, 4901–4909. 10.1038/onc.2011.187 21666710 PMC3175299

[B99] Mouta-Bellum C. Kirov A. Miceli-Libby L. Mancini M. L. Petrova T. V. Liaw L. (2009). Organ-specific lymphangiectasia, arrested lymphatic sprouting, and maturation defects resulting from gene-targeting of the PI3K regulatory isoforms p85alpha, p55alpha, and p50alpha. Dev. Dyn. 238, 2670–2679. 10.1002/dvdy.22078 19705443 PMC2826787

[B100] Muley A. Kim Uh M. Salazar-DE Simone G. Swaminathan B. James J. M. Murtomaki A. (2022). Unique functions for Notch4 in murine embryonic lymphangiogenesis. Angiogenesis 25, 205–224. 10.1007/s10456-021-09822-5 34665379 PMC9054879

[B101] Murakami M. Zheng Y. Hirashima M. Suda T. Morita Y. Ooehara J. (2008). VEGFR1 tyrosine kinase signaling promotes lymphangiogenesis as well as angiogenesis indirectly via macrophage recruitment. Arterioscler. Thromb. Vasc. Biol. 28, 658–664. 10.1161/ATVBAHA.107.150433 18174461

[B102] Muramatsu M. Yamamoto S. Osawa T. Shibuya M. (2010). Vascular endothelial growth factor receptor-1 signaling promotes mobilization of macrophage lineage cells from bone marrow and stimulates solid tumor growth. Cancer Res. 70, 8211–8221. 10.1158/0008-5472.CAN-10-0202 20924106

[B103] Nakayama H. Bruneau S. Kochupurakkal N. Coma S. Briscoe D. M. Klagsbrun M. (2015). Regulation of mTOR signaling by semaphorin 3F-neuropilin 2 interactions *in vitro* and *in vivo* . Sci. Rep. 5, 11789. 10.1038/srep11789 26156437 PMC4496725

[B104] Nandi P. Girish G. V. Majumder M. Xin X. Tutunea-Fatan E. Lala P. K. (2017). PGE2 promotes breast cancer-associated lymphangiogenesis by activation of EP4 receptor on lymphatic endothelial cells. BMC Cancer 17, 11. 10.1186/s12885-016-3018-2 28056899 PMC5217626

[B105] Niessen K. Zhang G. Ridgway J. B. Chen H. Kolumam G. Siebel C. W. (2011). The Notch1-Dll4 signaling pathway regulates mouse postnatal lymphatic development. Blood 118, 1989–1997. 10.1182/blood-2010-11-319129 21700774

[B106] Nurmi H. Saharinen P. Zarkada G. Zheng W. Robciuc M. R. Alitalo K. (2015). VEGF‐C is required for intestinal lymphatic vessel maintenance and lipid absorption. EMBO Mol. Med. 7, 1418–1425. 10.15252/emmm.201505731 26459520 PMC4644375

[B107] Olsson A. K. Dimberg A. Kreuger J. Claesson-Welsh L. (2006). VEGF receptor signalling - in control of vascular function. Nat. Rev. Mol. Cell Biol. 7, 359–371. 10.1038/nrm1911 16633338

[B108] Osborn A. J. Dickie P. Neilson D. E. Glaser K. Lynch K. A. Gupta A. (2015). Activating PIK3CA alleles and lymphangiogenic phenotype of lymphatic endothelial cells isolated from lymphatic malformations. Hum. Mol. Genet. 24, 926–938. 10.1093/hmg/ddu505 25292196

[B109] Pajusola K. Aprelikova O. Armstrong E. Morris S. Alitalo K. (1993). Two human FLT4 receptor tyrosine kinase isoforms with distinct carboxy terminal tails are produced by alternative processing of primary transcripts. Oncogene 8, 2931–2937. 7692369

[B110] Pajusola K. Aprelikova O. Korhonen J. Kaipainen A. Pertovaara L. Alitalo R. (1992). FLT4 receptor tyrosine kinase contains seven immunoglobulin-like loops and is expressed in multiple human tissues and cell lines. Cancer Res. 52, 5738–5743. 1327515

[B111] Pajusola K. Aprelikova O. Pelicci G. Weich H. Claesson-Welsh L. Alitalo K. (1994). Signalling properties of FLT4, a proteolytically processed receptor tyrosine kinase related to two VEGF receptors. Oncogene 9, 3545–3555. 7970715

[B112] Pan M. R. Chang T. M. Chang H. C. Su J. L. Wang H. W. Hung W. C. (2009). Sumoylation of Prox1 controls its ability to induce VEGFR3 expression and lymphatic phenotypes in endothelial cells. J. Cell Sci. 122, 3358–3364. 10.1242/jcs.050005 19706680

[B113] Park Y. G. Choi J. Jung H. K. Song I. K. Shin Y. Park S. Y. (2017). Fluid shear stress regulates vascular remodeling via VEGFR-3 activation, although independently of its ligand, VEGF-C, in the uterus during pregnancy. Int. J. Mol. Med. 40, 1210–1216. 10.3892/ijmm.2017.3108 28849193 PMC5593466

[B114] Park C. Lee J. Y. Yoon Y. S. (2011). Role of bone marrow-derived lymphatic endothelial progenitor cells for lymphatic neovascularization. Trends Cardiovasc Med. 21, 135–140. 10.1016/j.tcm.2012.04.002 22732548 PMC3384483

[B115] Parker M. W. Linkugel A. D. Goel H. L. Wu T. Mercurio A. M. Vander Kooi C. W. (2015). Structural basis for VEGF-C binding to neuropilin-2 and sequestration by a soluble splice form. Structure 23, 677–687. 10.1016/j.str.2015.01.018 25752543 PMC4394031

[B116] Planas-Paz L. Strilić B. Goedecke A. Breier G. Fässler R. Lammert E. (2012). Mechanoinduction of lymph vessel expansion. EMBO J. 31, 788–804. 10.1038/emboj.2011.456 22157817 PMC3280555

[B117] Porta C. Paglino C. Mosca A. (2014). Targeting PI3K/Akt/mTOR signaling in cancer. Front. Oncol. 4, 64. 10.3389/fonc.2014.00064 24782981 PMC3995050

[B118] Qin T. T. Xu G. C. Qi J. W. Yang G. L. Zhang K. Liu H. L. (2015). Tumour necrosis factor superfamily member 15 (Tnfsf15) facilitates lymphangiogenesis via up-regulation of Vegfr3 gene expression in lymphatic endothelial cells. J. Pathol. 237, 307–318. 10.1002/path.4577 26096340

[B119] Quentmeier H. Eberth S. Romani J. Weich H. A. Zaborski M. Drexler H. G. (2012). DNA methylation regulates expression of VEGF-R2 (KDR) and VEGF-R3 (FLT4). BMC Cancer 12, 19. 10.1186/1471-2407-12-19 22251800 PMC3297533

[B120] Ren B. Deng Y. Mukhopadhyay A. Lanahan A. A. Zhuang Z. W. Moodie K. L. (2010). ERK1/2-Akt1 crosstalk regulates arteriogenesis in mice and zebrafish. J. Clin. Invest. 120, 1217–1228. 10.1172/JCI39837 20237411 PMC2846043

[B121] Rofstad E. Galappathi K. Mathiesen B. (2014). Tumor interstitial fluid pressure—a link between tumor hypoxia, microvascular density, and lymph node metastasis. Neoplasia 16, 586–594. 10.1016/j.neo.2014.07.003 25117980 PMC4198829

[B122] Rondon-Galeano M. Skoczylas R. Bower N. I. Simons C. Gordon E. Francois M. (2020). MAFB modulates the maturation of lymphatic vascular networks in mice. Dev. Dyn. 249, 1201–1216. 10.1002/dvdy.209 32525258

[B123] Sakima M. Hayashi H. Mamun A. A. Sato M. (2018). VEGFR-3 signaling is regulated by a G-protein activator, activator of G-protein signaling 8, in lymphatic endothelial cells. Exp. Cell Res. 368, 13–23. 10.1016/j.yexcr.2018.04.007 29649427

[B124] Salameh A. Galvagni F. Bardelli M. Bussolino F. Oliviero S. (2005). Direct recruitment of CRK and GRB2 to VEGFR-3 induces proliferation, migration, and survival of endothelial cells through the activation of ERK, AKT, and JNK pathways. Blood 106, 3423–3431. 10.1182/blood-2005-04-1388 16076871

[B125] Sato M. Cismowski M. J. Toyota E. Smrcka A. V. Lucchesi P. A. Chilian W. M. (2006). Identification of a receptor-independent activator of G protein signaling (AGS8) in ischemic heart and its interaction with Gbetagamma. Proc. Natl. Acad. Sci. U. S. A. 103, 797–802. 10.1073/pnas.0507467103 16407149 PMC1334649

[B126] Shawber C. J. Funahashi Y. Francisco E. Vorontchikhina M. Kitamura Y. Stowell S. A. (2007). Notch alters VEGF responsiveness in human and murine endothelial cells by direct regulation of VEGFR-3 expression. J. Clin. Invest. 117, 3369–3382. 10.1172/JCI24311 17948123 PMC2030453

[B127] Siegfried G. Basak A. Cromlish J. A. Benjannet S. Marcinkiewicz J. Chrétien M. (2003). The secretory proprotein convertases furin, PC5, and PC7 activate VEGF-C to induce tumorigenesis. J. Clin. Invest. 111, 1723–1732. 10.1172/JCI17220 12782675 PMC156106

[B128] Simons M. Gordon E. Claesson-Welsh L. (2016). Mechanisms and regulation of endothelial VEGF receptor signalling. Nat. Rev. Mol. Cell Biol. 17, 611–625. 10.1038/nrm.2016.87 27461391

[B129] Souma T. Thomson B. R. Heinen S. Carota I. A. Yamaguchi S. Onay T. (2018). Context-dependent functions of angiopoietin 2 are determined by the endothelial phosphatase VEPTP. Proc. Natl. Acad. Sci. U. S. A. 115, 1298–1303. 10.1073/pnas.1714446115 29358379 PMC5819405

[B130] Spinella F. Caprara V. DI Castro V. Rosanò L. Cianfrocca R. Natali P. G. (2013). Endothelin-1 induces the transactivation of vascular endothelial growth factor receptor-3 and modulates cell migration and vasculogenic mimicry in melanoma cells. J. Mol. Med. Berl. 91, 395–405. 10.1007/s00109-012-0956-2 22965194

[B131] Spinella F. Garrafa E. DI Castro V. Rosanò L. Nicotra M. R. Caruso A. (2009). Endothelin-1 stimulates lymphatic endothelial cells and lymphatic vessels to grow and invade. Cancer Res. 69, 2669–2676. 10.1158/0008-5472.CAN-08-1879 19276384

[B132] Srinivasan R. S. Escobedo N. Yang Y. Interiano A. Dillard M. E. Finkelstein D. (2014). The Prox1-Vegfr3 feedback loop maintains the identity and the number of lymphatic endothelial cell progenitors. Genes Dev. 28, 2175–2187. 10.1101/gad.216226.113 25274728 PMC4180978

[B133] Stanczuk L. Martinez-Corral I. Ulvmar M. H. Zhang Y. Laviña B. Fruttiger M. (2015). cKit lineage hemogenic endothelium-derived cells contribute to mesenteric lymphatic vessels. Cell Rep. 10, 1708–1721. 10.1016/j.celrep.2015.02.026 25772358

[B134] Takada Y. Ye X. Simon S. (2007). The integrins. Genome Biol. 8, 215. 10.1186/gb-2007-8-5-215 17543136 PMC1929136

[B135] Takahashi T. Ueno H. Shibuya M. (1999). VEGF activates protein kinase C-dependent, but Ras-independent Raf-MEK-MAP kinase pathway for DNA synthesis in primary endothelial cells. Oncogene 18, 2221–2230. 10.1038/sj.onc.1202527 10327068

[B136] Takata Y. Kitami Y. Yang Z. H. Nakamura M. Okura T. Hiwada K. (2002). Vascular inflammation is negatively autoregulated by interaction between CCAAT/enhancer-binding protein-delta and peroxisome proliferator-activated receptor-gamma. Circ. Res. 91, 427–433. 10.1161/01.res.0000031271.20771.4f 12215492

[B137] Tammela T. Zarkada G. Wallgard E. Murtomäki A. Suchting S. Wirzenius M. (2008). Blocking VEGFR-3 suppresses angiogenic sprouting and vascular network formation. Nature 454, 656–660. 10.1038/nature07083 18594512

[B138] Taniguchi K. Kohno R. Ayada T. Kato R. Ichiyama K. Morisada T. (2007). Spreds are essential for embryonic lymphangiogenesis by regulating vascular endothelial growth factor receptor 3 signaling. Mol. Cell Biol. 27, 4541–4550. 10.1128/MCB.01600-06 17438136 PMC1900061

[B139] Uchida Y. James J. M. Suto F. Mukouyama Y. S. (2015). Class 3 semaphorins negatively regulate dermal lymphatic network formation. Biol. Open 4, 1194–1205. 10.1242/bio.012302 26319580 PMC4582121

[B140] Urner S. Planas-Paz L. Hilger L. S. Henning C. Branopolski A. Kelly-Goss M. (2019). Identification of ILK as a critical regulator of VEGFR3 signalling and lymphatic vascular growth. EMBO J. 38, e99322. 10.15252/embj.201899322 30518533 PMC6331728

[B141] Vo N. Goodman R. H. (2001). CREB-binding protein and p300 in transcriptional regulation. J. Biol. Chem. 276, 13505–13508. 10.1074/jbc.R000025200 11279224

[B142] Wang S. H. Chang J. S. Hsiao J. R. Yen Y. C. Jiang S. S. Liu S. H. (2017). Tumour cell-derived WNT5B modulates *in vitro* lymphangiogenesis via induction of partial endothelial-mesenchymal transition of lymphatic endothelial cells. Oncogene 36, 1503–1515. 10.1038/onc.2016.317 27593938

[B143] Wang C. Xu S. Tian Y. Ju A. Hou Q. Liu J. (2019). Lysyl oxidase-like protein 2 promotes tumor lymphangiogenesis and lymph node metastasis in breast cancer. Neoplasia 21, 413–427. 10.1016/j.neo.2019.03.003 30925417 PMC6439287

[B144] Wang Y. Nakayama M. Pitulescu M. E. Schmidt T. S. Bochenek M. L. Sakakibara A. (2010). Ephrin-B2 controls VEGF-induced angiogenesis and lymphangiogenesis. Nature 465, 483–486. 10.1038/nature09002 20445537

[B145] Weijts B. G. VAN Impel A. Schulte-Merker S. DE Bruin A. (2013). Atypical E2fs control lymphangiogenesis through transcriptional regulation of Ccbe1 and Flt4. PLoS One 8, e73693. 10.1371/journal.pone.0073693 24069224 PMC3771987

[B146] Wong B. W. Wang X. Zecchin A. Thienpont B. Cornelissen I. Kalucka J. (2017). The role of fatty acid β-oxidation in lymphangiogenesis. Nature 542, 49–54. 10.1038/nature21028 28024299

[B147] Wu B. Rockel J. S. Lagares D. Kapoor M. (2019). Ephrins and eph receptor signaling in tissue repair and fibrosis. Curr. Rheumatol. Rep. 21, 23. 10.1007/s11926-019-0825-x 30980212 PMC7112176

[B148] Wu H. Rahman H. N. A. Dong Y. Liu X. Lee Y. Wen A. (2018). Epsin deficiency promotes lymphangiogenesis through regulation of VEGFR3 degradation in diabetes. J. Clin. Invest. 128, 4025–4043. 10.1172/JCI96063 30102256 PMC6118634

[B149] Xu Y. Yuan L. Mak J. Pardanaud L. Caunt M. Kasman I. (2010). Neuropilin-2 mediates VEGF-C-induced lymphatic sprouting together with VEGFR3. J. Cell Biol. 188, 115–130. 10.1083/jcb.200903137 20065093 PMC2812843

[B150] Yang Y. Cha B. Motawe Z. Y. Srinivasan R. S. Scallan J. P. (2019). VE-cadherin is required for lymphatic valve formation and maintenance. Cell Rep. 28, 2397–2412. 10.1016/j.celrep.2019.07.072 31461654 PMC6743082

[B151] Yang Y. García-Verdugo J. M. Soriano-Navarro M. Srinivasan R. S. Scallan J. P. Singh M. K. (2012). Lymphatic endothelial progenitors bud from the cardinal vein and intersomitic vessels in mammalian embryos. Blood 120, 2340–2348. 10.1182/blood-2012-05-428607 22859612 PMC3447786

[B152] Yin X. Johns S. C. Lawrence R. Xu D. Reddi K. Bishop J. R. (2011). Lymphatic endothelial heparan sulfate deficiency results in altered growth responses to vascular endothelial growth factor-C (VEGF-C). J. Biol. Chem. 286, 14952–14962. 10.1074/jbc.M110.206664 21343305 PMC3083229

[B153] Yoo H. Lee Y. J. Park C. Son D. Choi D. Y. Park J.-H. (2020). Epigenetic priming by Dot1l in lymphatic endothelial progenitors ensures normal lymphatic development and function. Cell Death and Dis. 11, 14. 10.1038/s41419-019-2201-1 PMC6944698 31908356

[B154] Yoshida S. Hamuy R. Hamada Y. Yoshimoto H. Hirano A. Akita S. (2015). Adipose-derived stem cell transplantation for therapeutic lymphangiogenesis in a mouse secondary lymphedema model. Regen. Med. 10, 549–562. 10.2217/rme.15.24 26237700

[B155] Yoshimatsu Y. Miyazaki H. Watabe T. (2016). Roles of signaling and transcriptional networks in pathological lymphangiogenesis. Adv. Drug Deliv. Rev. 99, 161–171. 10.1016/j.addr.2016.01.020 26850127

[B156] Yu J. Zhang X. Kuzontkoski P. M. Jiang S. Zhu W. Li D. Y. (2014). Slit2N and Robo4 regulate lymphangiogenesis through the VEGF-C/VEGFR-3 pathway. Cell Commun. Signal 12, 25. 10.1186/1478-811X-12-25 24708522 PMC4122147

[B157] Zampell J. C. Yan A. Avraham T. Daluvoy S. Weitman E. S. Mehrara B. J. (2012). HIF-1α coordinates lymphangiogenesis during wound healing and in response to inflammation. Faseb J. 26, 1027–1039. 10.1096/fj.11-195321 22067482 PMC3470728

[B158] Zhang C. Zhu M. Wang W. Chen D. Chen S. Zheng H. (2019). TNF-α promotes tumor lymph angiogenesis in head and neck squamous cell carcinoma through regulation of ERK3. Transl. Cancer Res. 8, 2439–2448. 10.21037/tcr.2019.09.60 35116996 PMC8798551

[B159] Zhang L. Zhou F. Han W. Shen B. Luo J. Shibuya M. (2010). VEGFR-3 ligand-binding and kinase activity are required for lymphangiogenesis but not for angiogenesis. Cell Res. 20, 1319–1331. 10.1038/cr.2010.116 20697430

[B160] Zhang X. Groopman J. E. Wang J. F. (2005). Extracellular matrix regulates endothelial functions through interaction of VEGFR-3 and integrin alpha5beta1. J. Cell Physiol. 202, 205–214. 10.1002/jcp.20106 15389531

[B161] Zheng W. Tammela T. Yamamoto M. Anisimov A. Holopainen T. Kaijalainen S. (2011). Notch restricts lymphatic vessel sprouting induced by vascular endothelial growth factor. Blood 118, 1154–1162. 10.1182/blood-2010-11-317800 21566091

[B162] Zhou H. J. Chen X. Huang Q. Liu R. Zhang H. Wang Y. (2014). AIP1 mediates vascular endothelial cell growth factor receptor-3-dependent angiogenic and lymphangiogenic responses. Arterioscler. Thromb. Vasc. Biol. 34, 603–615. 10.1161/ATVBAHA.113.303053 24407031 PMC3952062

[B163] Zhou B. Si W. Su Z. Deng W. Tu X. Wang Q. (2013). Transcriptional activation of the Prox1 gene by HIF-1α and HIF-2α in response to hypoxia. FEBS Lett. 587, 724–731. 10.1016/j.febslet.2013.01.053 23395615

[B164] Zhuo W. Luo C. Wang X. Song X. Fu Y. Luo Y. (2010). Endostatin inhibits tumour lymphangiogenesis and lymphatic metastasis via cell surface nucleolin on lymphangiogenic endothelial cells. J. Pathol. 222, 249–260. 10.1002/path.2760 20814900

